# Biodiversity census of Lake St Lucia, iSimangaliso Wetland Park (South Africa): Gastropod molluscs

**DOI:** 10.3897/zookeys.440.7803

**Published:** 2014-09-15

**Authors:** Renzo Perissinotto, Nelson A.F. Miranda, Jacqueline L. Raw, Nasreen Peer

**Affiliations:** 1 DST/NRF Research Chair in Shallow Water Ecosystems, Nelson Mandela Metropolitan University, P.O. Box 77000, Port Elizabeth 6031 South Africa; 2 School of Life Sciences, University of KwaZulu-Natal, P Bag X54001, Durban 4001 South Africa

**Keywords:** Mollusca, Gastropoda, biodiversity census, hypersalinity, iSimangaliso Wetland Park, illustrated checklist

## Abstract

The recent dry phase experienced by the St Lucia estuarine system has led to unprecedented desiccation and hypersaline conditions through most of its surface area. This has changed only recently, at the end of 2011, with the onset of a new wet phase that has already caused a major shift to oligo- and mesohaline conditions. The estuary mouth, however, remains closed to the ocean, making the weak connection recently established between the St Lucia and the Mfolozi estuaries the only conveyance for marine recruitment. As a result, only 10 indigenous and two alien aquatic gastropod species are currently found living in the St Lucia estuarine lake. This is out of a total of 37 species recorded within the system since the earliest survey undertaken in 1924, half of which have not been reported in the literature before. The tick shell, *Nassarius kraussianus*, which was consistently found in large abundance prior to the recent dry phase, appears to have temporarily disappeared from the system, probably as a result of the extinction of *Zostera* marine grasses inside the lake. Population explosions of the bubble shell *Haminoea natalensis*, with its distinct egg masses, were recorded seasonally until 2009, but the species has subsequently not been observed again. A molecular DNA analysis of the various populations previously reported as belonging to the same assimineid species, variably referred to as *Assiminea capensis*, *A. ovata*, or *A. bifasciata*, has revealed that the St Lucia assemblage actually comprises two very distinct taxa, *A.* cf. *capensis* and a species provisionally referred to here as “*A.*” aff. *capensis* or simply Assimineidae sp. In the mangroves, the climbing whelk *Cerithidea decollata* is still found in numbers, while ellobiids such as *Cassidula labrella*, *Melampus semiaratus* and *M. parvulus* are present in low abundances and all previously recorded littorinids have disappeared. A number of alien freshwater species have colonized areas of the system that have remained under low salinity. These include the invasive thiarid *Tarebia granifera*, which can be found in concentrations exceeding 5000 ind.m^-2^, the lymnaeid *Pseudosuccinea columella* and the physid *Aplexa marmorata*.

## Introduction

Lake St Lucia is a large, complex estuarine lake situated on the South African east coast. It has been extensively investigated since the late 1940s, as it is the largest such system in Africa, the oldest protected estuary in the world and a Ramsar Wetland of International Importance since 1986 (Porter 2013). It currently forms a crucial part of the iSimangaliso Wetland Park, which is South Africa’s first UNESCO World Heritage Site. During the past century, the system has undergone a number of changes related to anthropogenic activities, which superimposed on an already complex and variable climate have escalated the magnitude of its regular shifts from wet to dry states (Perissinotto et al. 2013). Typically the system experiences sub-decadal alternations of droughts and anomalous wet conditions, at times resulting in severe floods. Recently, the system has undergone a major shift, from a prolonged dry phase during 2002–2011 to flood conditions during 2012–2013, which has resulted in the current predominance of oligohaline conditions through most of its basins (Raw et al. 2013; Peer et al. 2014).

The rich biodiversity of the St Lucia estuarine lake is one of the main drivers of its special conservation status. Species are the building-blocks of any ecosystem, yet in the St Lucia case there are many misidentifications and several groups of invertebrates remain poorly investigated or completely ignored. A few detailed taxonomic studies of selected invertebrate groups have already been undertaken, starting from 2010, using a combination of traditional morphological analyses and molecular DNA barcoding. These have consistently revealed the occurrence of species that were either previously confused with others or completely unknown to science (e.g. Daly et al. 2012; Gómez et al. 2012; Carrasco and Perissinotto 2012; Todaro et al. 2011, 2013). A systematic approach has been implemented recently, aimed at producing a modern “Biodiversity Census” for the estuarine lake. The main objective of the initial phase of the census is to add accurate checklists of as many aquatic invertebrate groups as possible to those already existing for the vertebrates and the macrophytes. As the diversity census of bivalve molluscs and true crabs have already been completed and published (Nel et al. 2012; Peer et al. 2014), this new contribution focused on the gastropod molluscs is regarded as the third of the series planned within the census.

The class Gastropoda is the most diverse among the molluscs and includes about 55000 extant aquatic species globally (Brusca and Brusca 2003). Gastropods play an integral part in the functioning of aquatic ecosystems (Carlén and Ólafsson 2002). In estuarine ecosystems such as St Lucia, gastropods are important components of food webs and energy pathways. They are mostly detritivores or herbivores, feeding on a large variety of decomposing organic materials or on benthic or epiphytic microalgae, protozoans, bacteria and fungi. There are also a few predatory or scavenger species, especially among the muricids, nassariids and naticids. Gastropods also play a key role in nutrient dynamics in mangrove ecosystems, where they graze on encrusting fauna and microalgae, thus cleaning the pneumatophores (Ghasemi et al. 2011). In turn, both snails and limpets are consumed by a variety of predators, including birds (Whitfield and Cyrus 1978; Hockey et al. 2005; Turpie et al. 2013), fish (Blaber and Blaber 1980; Whitfield 1998; Dyer et al. 2013), crabs (Sousa 1993; Schaefer and Zimmer 2013), leeches, larvae of marsh flies and aquatic beetles (Appleton 1996), as well as anemones (Daly et al. 2012). Some freshwater species living on the fringes of Lake St Lucia, or even entering the estuarine system in times of freshwater dominance, may be of veterinarian/medical importance as they act as vectors of parasites responsible for water-borne diseases, such as bilharzia, fascioliasis and paramphistomiasis (Appleton 1996). In estuaries, the diversity of gastropods is determined to a large extent by changes in physico-chemical conditions and the availability of detritus and microalgae as their primary food sources (Carlén and Ólafsson 2002).

The purpose of this study is thus to provide a comprehensive review of the diversity of gastropod molluscs in the St Lucia estuarine lake. This includes identifying species that are currently present in the system and comparing them with what was collected in past surveys. Changes in diversity over time are related to shifts in environmental and climatic conditions that have occurred during the past century. The compilation of an annotated and illustrated checklist of all gastropod species recorded so far within the system is designed to aid managers, researchers and visitors in the iSimangaliso Wetland Park with the identification of these important molluscs.

## Methods

**Figure 1.**
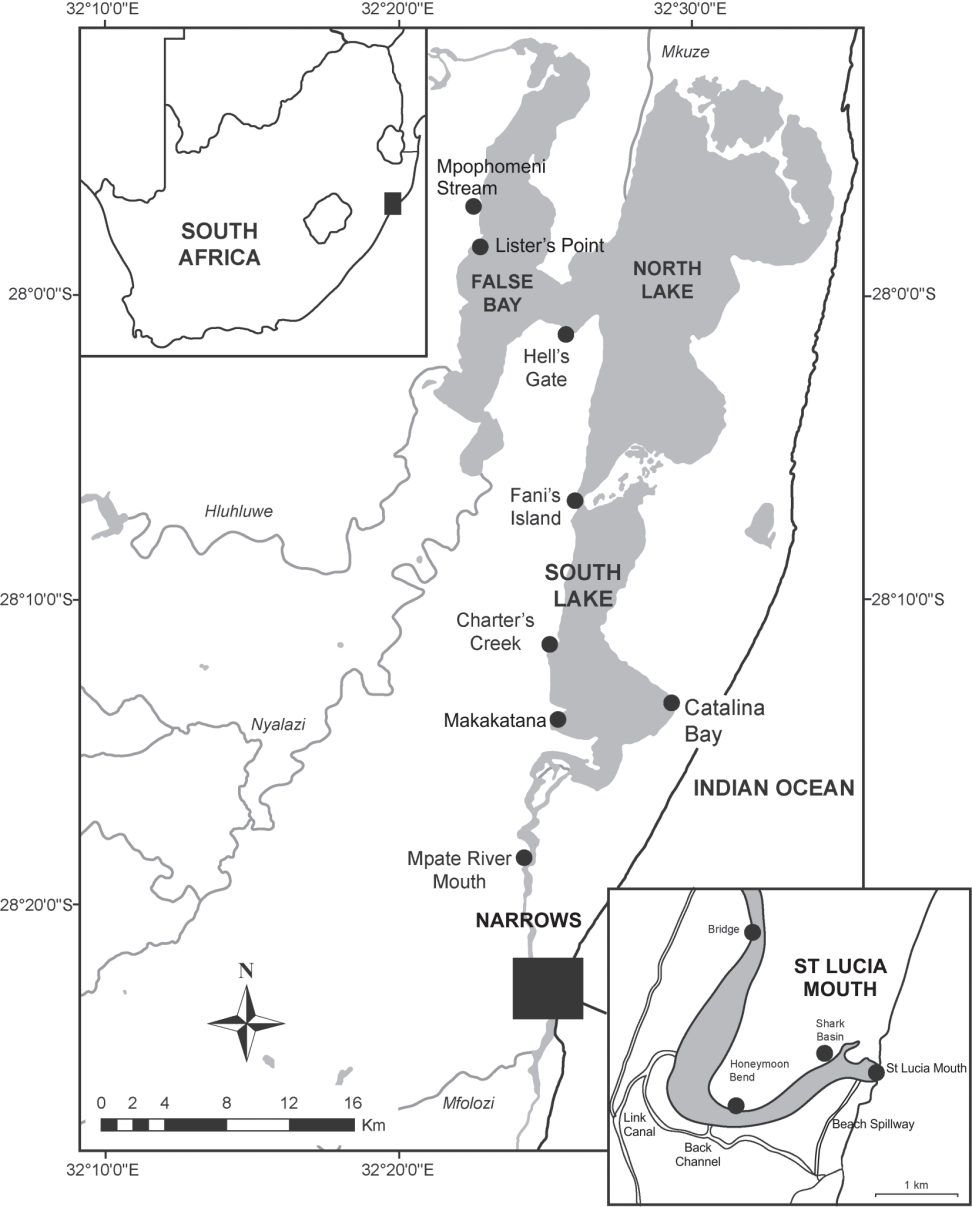
Map of the St Lucia estuarine lake, with position of main collection sites used in the study. Adapted from Peer et al. (2014).

The St Lucia estuarine lake is located on the north coast of KwaZulu-Natal, between 27°52' to 28°24'S and 32°21' to 32°34'E. The system has a surface area of approximately 350 km^2^ (Taylor et al. 2006), with a perimeter of approximately 347 km at low water and depth of 0.9 m (Cyrus et al. 2011). It is subdivided into three lake basins, viz. False Bay, North and South lakes, which communicate with the mouth via a narrow channel known as “The Narrows” (Figure 1).

The first gastropod records from the St Lucia system date back to 1924, with specimens reposited in the KwaZulu-Natal Museum (NMSA), in Pietermaritzburg, ever since. Further collections were later undertaken during the two surveys of the University of Cape Town, in 1948–1949 and 1964–1965. Specimens collected during these surveys are currently reposited at the Iziko South African Museum (ISAM), in Cape Town. In 1982-1983, a dedicated collecting survey was undertaken throughout the lake system by the provincial conservation authority, the Natal Parks Board (NPB). The same authority, renamed Ezemvelo KwaZulu-Natal Wildlife (EKZNW), completed another similar survey during 2005, at the peak of the most recent drought. A number of publications containing gastropod records have also been published since 1954, mainly by researchers operating at the universities of KwaZulu-Natal, Zululand, Rhodes and Cape Town (e.g. Day et al. 1954; Millard and Broekhuysen 1970; Boltt 1975; Pillay and Perissinotto 2008, 2013; MacKay et al. 2010). Finally, a dedicated survey during 2012 and 2013 was conducted as part of this study, in order to establish the current status of gastropod diversity within the system.

### Museum and literature data

Specimens and data of gastropods collected at St Lucia in past surveys were obtained from the KwaZulu-Natal Museum in Pietermaritzburg and the Iziko South African Museum in Cape Town. Particularly rich collections from St Lucia were undertaken by the NMSA in 1987. Reference to specimens from either museum are here complemented with their accession numbers. Literature involving past macrobenthic surveys undertaken in the St Lucia Estuary (e.g. Day et al. 1954; Millard and Broekhuysen 1970; Boltt 1975; Weerts 1993; Pillay and Perissinotto 2008; MacKay et al. 2010) were reviewed to obtain information about gastropod diversity and the environmental conditions in which species were collected. Information such as synonyms, common name, size distribution and other records about the various species were used to annotate the illustrated checklist.

### Historical surveys

Both NPB 1982–1983 and EKZNW 2005 collecting surveys were conducted at the onset of extreme drought conditions, when organisms within the estuarine lake were experiencing mass kills in response to hypersaline conditions and lake fragmentation/desiccation. Dead gastropods were mainly found washed up along the shorelines of St Lucia. The Natal Parks Board surveyed the banks of the whole lake from December 1982 to April 1983, collecting freshly dead specimens. These were later identified to species level by the late R.N. Kilburn. Ezemvelo KZN Wildlife surveyed the St Lucia banks in 2005, taking samples at fixed points along a number of transects in both South and North lakes (Figure 1). On this occasion, gastropods were identified by D. Herbert and R.N. Kilburn. In all cases, no specialized equipment was used and specimens were collected by hand at the surface or within the sediment by using spades and/or mechanical grabs.

### 2012–2013 survey

Two surveys were conducted in March and July 2012. The survey in March was conducted at Fani’s Island, St Lucia Mouth, Hell’s Gate, Makakatana, Lister’s Point, the Bridge over the Narrows and along a transect from Catalina Bay to Charter’s Creek. The Back Channel, Shark Basin and Mpate Mouth were also visited (Figure 1, Table 1). Macrobenthic samples were taken using a Zabalocki-type Eckman grab with a sampling area of 0.0236 m^2^ and depth of 15 cm. Each sample was made up of three grabs, with three replicate samples taken at each site. Replicate samples were emptied into 20 L buckets and water was added in each sample. Each sample was stirred vigorously to suspend benthic organisms and the supernatant was passed through a 500 µm sieve. This procedure was repeated five times for each replicate sample. Material that was retained on the sieve was emptied into plastic jars. Sediments that were left in the bucket were washed through a 2 mm sieve. Samples were preserved in 4% phloxin-stained formalin. Qualitative samples were also collected with a D-net. At each site, the net was pushed over the sediment surface for a distance of approximately 5 m. At least one D-net sample was collected from each site. Macrofauna retained on the D-net were emptied into plastic jars and 4% phloxin-stained formalin was added. Both sampling methods (grab and D-net) were used at Fani’s Island, St Lucia Mouth, Hell’s Gate, Makakatana and along a transect from Catalina Bay to Charter's Creek, while only D-net samples were collected from the Bridge and the Back Channel (Figure 1, Table 1).

**Table 1.** List of localities mentioned in the study with their coordinates and key biophysical characteristics.

Region	Site name	Latitude, Longitude	Comments
False Bay	Lister’s Point	-27.9697, 32.3847	Muddy and fossiliferous coquina substrate; sparse macrophyte cover.
	Mpophomeni Stream	-27.9519, 32.3771	Brackish forest stream with muddy sand substrate.
North Lake	Hell’s Gate	-28.0118, 32.4438	Muddy and fossiliferous coquina substrate; sparse macrophyte cover.
South Lake	Catalina Bay	-28.2237, 32.4839	Limestone flat, muddy sand substrate; freshwater seepage from dune aquifers; exhibiting sedges such as *Phragmites*, *Juncus* and *Schoenoplectus*.
	Charter’s Creek	-28.1994, 32.4162	Muddy sand substrate; submerged macrophytes such as *Ruppia* sp. frequently recorded.
	Fani’s Island	-28.1091, 32.4341	Muddy sand substrate; historic presence of *Zostera capensis* from this site southwards.
	Makakatana	-28.2364, 32.4199	Sandy substrate, brackish conditions and relatively low turbidity; submerged macrophytes such as *Ruppia cirrhosa* frequently recorded.
Narrows	Mpate River Mouth	-28.2945, 32.4012	Muddy substrate, fringed by intertidal reeds *Phragmites australis* and mangroves.
	St Lucia Bridge	-28.3689, 32.4096	Muddy substrate, fringed by mangroves *Avicennia marina* and *Bruguiera gymnorrhiza* and some *Hibiscus tiliaceus*; submerged macrophytes such as *Stuckenia pectinata* generally present.
St Lucia Mouth	Honeymoon Bend	-28.3871, 32.4032	Tidal influence; muddy substrate, fringed by *Phragmites* reeds and mangroves *Avicennia marina* and *Bruguiera gymnorrhiza*.
	Mfolozi Back Channel	-28.3922, 32.4094	
	Mfolozi Link Canal	-28.3945, 32.3943	
	Mfolozi-St Lucia Beach Spillway	-28.3892, 32.4238	Sandy substrate; recent shallow link between St Lucia Mouth and Mfolozi River; influenced by tide.
	Shark Basin	-28.3831, 32.4203	Sandy mud substrate; fringed by reeds and sedges as well as mangroves; influenced by tides and freshwater draining from adjacent areas to the north.

The survey also included the manual collection of dead gastropod shells along the shoreline of the lake, within close proximity of the sampling stations. Further collections on an opportunistic basis were undertaken throughout 2013. In the laboratory, each sample was emptied into a sorting tray and gastropods were separated and identified with the aid of specialized literature and, where necessary, external taxonomy experts. Suitable specimens and shells were photographed in a standardized way, so as to show morphological characteristics that aid in their identification in an illustrated checklist.

## Results

A total of 20 families and 37 species of gastropods have been found in the St Lucia estuarine system since 1924, with half of the species not previously recorded in the literature. These include *Afrolittorina africana*, *Alaba pinnae*, *Bulinus natalensis*, *Cerithium dialeucum*, *Ergalatax heptagonalis*, *Jujubinus suarezensis*, *Littoraria coccinea glabrata*, *Littoraria intermedia*, *Littoraria pallescens*, *Littoraria subvittata*, *Lymnaea natalensis*, *Melampus parvulus*, *Murex brevispinus*, *Neritina gagates*, *Neritina natalensis*, *Phalium areola*, *Pterotrachea* cf. *hippocampus* and *Purpura bufo* (Table 2, Appendix).

**Table 2.** Gastropod species originally recorded from the St Lucia estuarine lake. Reference codes: B: Boltt (1975); BKJC: Blaber, Kure, Jackson and Cyrus (1983); DMB: Day, Millard and Broekhuysen (1954); EKW: Ezemvelo KwaZulu-Natal Wildlife Survey Record (2005); ISAM: Iziko South African Museum Collection Record (Accession No.); NMSA: KwaZulu-Natal Museum Collection Record (Accession No.); MB: Millard and Broekhuysen (1970); MCR: MacKay, Cyrus and Russell (2010); MPA1: Miranda, Perissinotto and Appleton (2010); MPA2: Miranda, Perissinotto and Appleton (2011); MET: Miranda et al. (2014), NMPO: Nelson Miranda Personal Observation (2013); NPB: Natal Parks Board Survey Record (1982/83, 1988); OF: Owen and Forbes (1997); PMRP: Recorded During This Study (2014); PP: Pillay and Perissinotto (2008); RMP: Raw, Miranda and Perissinotto (2013); V: Vrdoljak (2004); W: Weerts (1993). The classification scheme follows Bouchet and Rocroi (2005). (* = New record for Lake St Lucia).

Species (original record)	Current valid name	Record year(s)	Reference(s)
**TROCHIDAE**			
*Jujubinus suarezensis* (P. Fischer, 1878)	Idem*	1987, Jul–Nov 2012	NMSA (E2145), PMRP 2014
**NERITIDAE**			
*Neritina gagates* Lamarck, 1822	Idem*	1988	NPB 1988
*Neritina natalensis* Reeve, 1855	Idem*	1924, 1987	NMSA (B7378, D5947)
**THIARIDAE**			
*Melanoides tuberculata* (Müller, 1774)	Idem	Jul 2012–Nov 2013	RMP 2013
*Tarebia granifera* (Lamarck, 1822)	Idem	2006, Apr–Jul 2007, Feb 2007–Mar 2011, 2010	PP 2008, MPA 2010, MPA 2011, NMSA (W8287)
**CERITHIIDAE**			
*Cerithium dialeucum* Philippi, 1849	Idem*	Nov 2013	PMRP 2014
**LITIOPIDAE**			
*Alaba pinnae* (Krauss, 1848)	Idem*	1967, 1987	NMSA (8083, E2164)
**POTAMIDIDAE**			
*Cerithidea decollata* (Linnaeus, 1767)	Idem	Jul 1948–Jul 1951, Jul 1964 & Jan 1965, 2011–2013	NMSA (A6384), ISAM (STL60), DMB 1954, MB 1970, PMRP 2014
**LITTORINIDAE**			
*Afrolittorina africana* (Krauss in Philippi, 1847)	Idem*	1971	NMSA (A1635)
*Littoraria glabrata* (Philippi, 1846)	*Littoraria coccinea glabrata* (Philippi, 1846)*	1971	NMSA (A1636)
*Littoraria intermedia* (Philippi, 1846)	Idem*	1971, 1987, Mar 2012	NMSA (A1634, D9983, E460), PMRP 2014
*Littoraria pallescens* (Philippi, 1846)	Idem*	1987	NMSA (D9980)
*Littoraria subvittata* (Reid, 1986)	Idem*	Not reported (*sine die*)	NMSA (7128)
*Littoraria scabra* (Linnaeus, 1758)	Idem	Jul 1948–Jul 1951, Jul 1964 & Jan 1965, Jan–Jul 1972 & Jan 1973	ISAM (STL50B), DMB 1954, MB 1970, B 1975
**PTEROTRACHEIDAE**			
*Pterotrachea* cf. *hippocampus* Philippi, 1836	Idem*	Aug 2013	PMRP 2014
**ASSIMINEIDAE**			
*Assiminea* sp. Fleming, 1828	Probably comprising both *Assiminea* cf. *capensis* and Assimineidae sp.	Jul 1948–Jul 1951, Aug 1981–Jul 1982, Oct 2005	DMB 1954, BKJC 1983, PP 2008
*Assiminea bifasciata* Nevill, 1880	*Assiminea* cf. *capensis* Bartsch, 1915	Jul 1964 & Jan 1965, Jan–Jul 1972 & Jan 1973	ISAM (STL104G), MB 1970, B 1975
*Assiminea durbanensis*	Assimineidae sp. or “*Assiminea*” aff. *capensis* (Sowerby, 1892)*	Jan & May 1992	W 1993
*Assiminea* cf. *ovata* (Krauss, 1848)	*Assiminea* cf. *capensis* Bartsch, 1915	2007–2009	MPA2 2011
*Assiminea* cf. *capensis* Bartsch, 1915	Idem	Jan 1927, 1987, 2011-2013	NMSA (1987), RMP 2013, WPPC 2014
*Coriandria durbanensis* (Tomlin, 1916)	Assimineidae sp. or “*Assiminea*” aff. *capensis* (Sowerby, 1892)*	Jul 1948–Jul 1951, Jul 2012-May 2013	ISAM (STL18A), DMB 1954, RMP 2013, MET 2014
*Syncera* sp. Gray, 1821	*Nomen nudum*; probably confused with *Assiminea* sp.	Jul 1948, Jul 1964 & Jan 1965	ISAM (STL64A), MB 1970
**CASSIDAE**			
*Phalium areola* (Linnaeus, 1758)	Idem*	Not reported (*sine die*)	NMSA (B4786)
**NASSARIIDAE**			
*Nassa kraussiana* (Dunker, 1846)	*Nassarius kraussianus* (Dunker, 1846)	Jul 1964 & Jan 1965	MB 1970
*Nassarius kraussianus* (Dunker, 1846)	Idem	Jul 1948–Jul 1951, Feb 1971, Dec 1972, Jan–Jul 1972 & Jan 1973, Dec 1981, Aut 2005 & Spr 2006	NMSA (B5533, W1752, 9144), ISAM (STL6C), DMB 1954, B 1975, MCR 2010
**MURICIDAE**			
*Ergalatax heptagonalis* (Reeve, 1846)	Idem*	1987	NMSA (D5772)
*Murex brevispinus* Lamarck, 1822	Idem*	Jul-Nov 2012	PMRP 2014
*Purpura bufo* Lamarck, 1822	Idem*	Jul 2013	PMRP 2014
**HAMINOEIDAE**			
*Cylichna africana* Bartsch, 1915	*Haminoea natalensis* (Krauss, 1848) or *Cylichna tubulosa* Gould, 1859	Jul 1972 & Jan 1973	B 1975
*Haminea gracilis* (Sowerby, III 1897)	*Haminoea natalensis* (Krauss, 1848)	Jul 1964 & Jan 1965	MB 1970
*Haminoea natalensis* (Krauss, 1848)	Idem	Jul 1948–Jul 1951, Dec 1962, Apr 1963, Apr 1965, Jun 1987, 2006, 2007	NMSA (A2362, A2228, D9971, E478), ISAM (STL6B), DMB 1954, PP 2008, MPA2 2011
*Haminea petersi* (Martens, 1879)	*Haminoea natalensis* (Krauss, 1848)	Aug 1981–Jul 1982	BKJC 1983
**APLYSIIDAE**			
*Barnardaclesia cirrhifera* (Quoy & Gaimard, 1832)	*Bursatella leachii* Blainville, 1817	Jul 1948-Jul 1951	DMB 1954
*Notarchus cirrhifera* (Quoy & Gaimard, 1832)	*Bursatella leachii* Blainville, 1817	Jul 1964 & Jan 1965	MB 1970
*Stilocheilus striatus* (Quoy & Gaimard, 1832)	Idem	May 2007	MPA2 2011
**SIPHONARIIDAE**			
*Siphonaria oculus* Krauss, 1848	Idem	Jul 1948–Jul 1951, Jul 1964 & Jan 1965	ISAM (STL43A), DMB 1954, MB 1970
**PLANORBIDAE**			
*Bulinus natalensis* (Krauss in Küster, 1841)	Idem*	Nov 2012	PMRP 2014
*Bulinus tropicus* (Krauss, 1848)	Idem	Jul 1948, Jul 1964 & Jan 1965, May 2002–Apr 2003	ISAM (STL104G), DMB 1954, MB 1970, PM 2013; VMT May 2002-Apr 2003
*Bulinus forskalii* (Ehrenberg, 1831)	Idem	May 2002-Apr 2003	V 2004
**PHYSIDAE**			
*Aplexa marmorata* (Guilding, 1828)	Idem	Aug 2009, 2009–2010	MPA1 2010, MPA2 2011
**LYMNAEIDAE**			
*Lymnaea natalensis* (Krauss, 1848)	Idem*	1982–1983	NPB 1982-1983
*Pseudosuccinea columella* (Say, 1817)	Idem	Aug 2009	MPA1 2010
**SUCCINEIDAE**			
*Oxyloma patentissima* (Menke in Pfeiffer, 1853)	Idem	May 2002–Apr 2003	V 2004
**ELLOBIIDAE**			
*Cassidula labrella* (Deshayes, 1830)	Idem	Jul 1964 & Jan 1965	ISAM (STL237M), MB 1970, PMRP 2014
*Melampus ordinarius* Melvill & Ponsonby, 1901	*Melampus lividus* (Deshayes, 1830)	Jul 1964 & Jan 1965	ISAM (STL237Y), MB 1970
*Melampus parvulus* Pfeiffer, 1856	Idem*	2012–2013	PMRP 2014
*Melampus semiaratus* Connolly, 1912	Idem	Jul 1964 & Jan 1965, Mar 2012	ISAM (STL237N), MB 1970

The earliest records of gastropod specimens collected in the St Lucia system are from the KwaZulu-Natal Museum (NMSA) and date back as far as 1924. Seventeen species originating from St Lucia are currently reposited in its collections, mostly obtained during dedicated surveys conducted in 1987 (Table 2). Of these, 11 are among the new records reported here, as they were never included in previous reports or publications on Lake St Lucia. Another two of the new records were collected during surveys undertaken by the provincial conservation authority, EKZNW, while the other six were only revealed during the latest survey of 2012–2013. Although three among the latter were only recorded as dead shells (i.e. *Cerithium dialeucum*, *Murex brevispinus*, *Purpura bufo*), they were found in sufficient number and deep enough in the upper reaches of the estuarine lake to suggest with reasonable confidence they were once established within the system, rather than accidentally introduced there.

**Figure 2.**
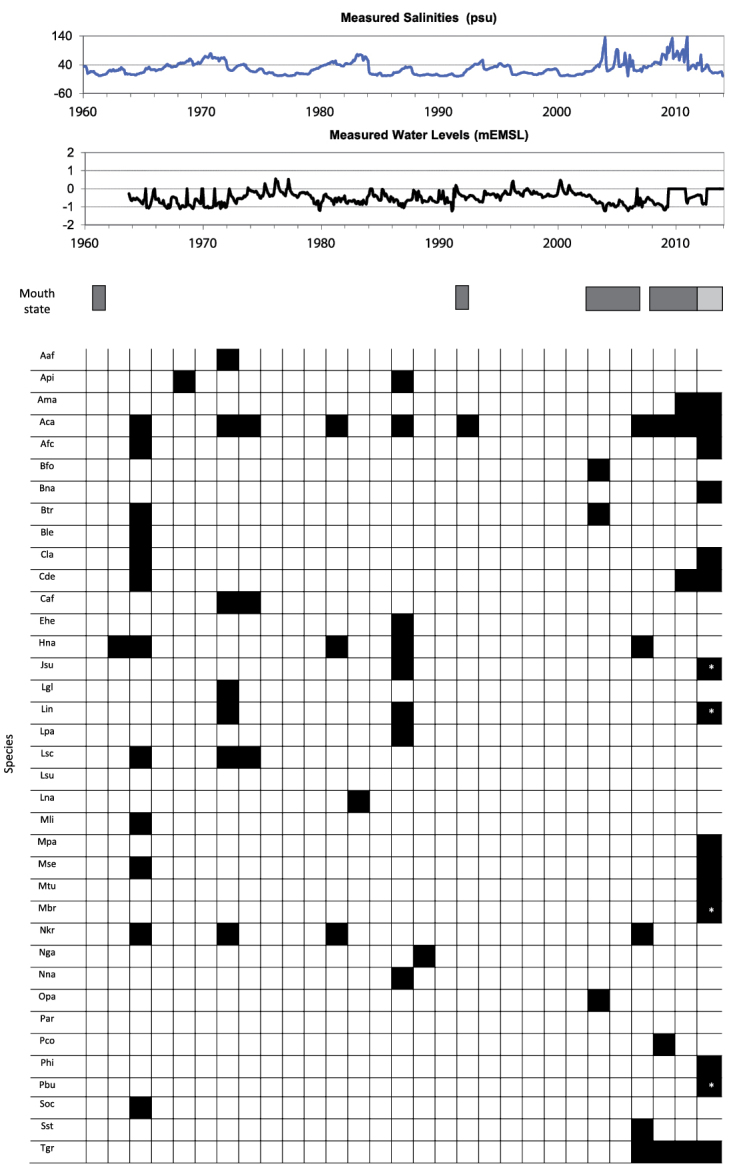
Records of gastropod species collected at Lake St Lucia in relation to changes in salinity, water levels and mouth state during the period 1960-present. Dark gray bar indicates closed mouth, light gray bar indicates intermittent connection with the ocean. No continuous physico-chemical measurements are available for the period prior to 1960. Species codes: Aaf: *Afrolittorina africana*; Api: *Alaba pinnae*; Ama: *Aplexa marmorata*; Aca: *Assiminea* cf. *capensis*; Afc: Assimineidae sp. (“*Assiminea*” aff. *capensis*); Bfo: *Bulinus forskalii*; Bna: *Bulinus natalensis*; Btr: *Bulinus tropicus*; Ble: *Bursatella leachii*; Cla: *Cassidula labrella*; Cde: *Cerithidea decollata*; Cdi: *Cerithium dialeucum*; Ehe: *Ergalatax heptagonalis*; Hna: *Haminoea natalensis*; Jsu: *Jujubinus suarezensis*; Lgl: *Littoraria coccinea glabrata*; Lin: *Littoraria intermedia*; Lpa: *Littoraria pallescens*; Lsc: *Littoraria scabra*; Lsu: *Littoraria subvittata*; Lna: *Lymnaea natalensis*; Mli: *Melampus lividus*; Mpa: *Melampus parvulus*; Mse: *Melampus semiaratus*; Mtu: *Melanoides tuberculata*; Mbr: *Murex brevispinus*; Nkr: *Nassarius kraussianus*; Nga: *Neritina gagates*; Nna: *Neritina natalensis*; Opa: *Oxyloma patentissima*; Par: *Phalium areola*; Pco: *Pseudosuccinea columella*; Phi: *Pterotrachea* cf. *hippocampus*; Pbu: *Purpura bufo*; Soc: *Siphonaria oculus*; Sst: *Stylocheilus striatus*; Tgr: *Tarebia granifera*.

Only 12 species were found alive within the system during the recent 2012–2013 survey. These include *Aplexa marmorata*, *Assiminea* cf. *capensis*, Assimineidae sp., *Bulinus natalensis*, *Cassidula labrella*, *Cerithidea decollata*, *Lymnaea natalensis*, *Melampus parvulus*, *Melampus semiaratus*, *Melanoides tuberculata*, *Pterotrachea* cf. *hippocampus* and *Tarebia granifera* (Figure 2). Among them, five are freshwater dwellers (*Aplexa marmorata*, *Bulinus natalensis*, *Lymnaea natalensis*, *Melanoides tuberculata* and *Tarebia granifera*) that have entered the system only recently, in response to the establishment of a new wet phase after the prolonged drought of 2002–2011. *Melanoides tuberculata* was found in high abundance at Shark Basin, at shallow depths in permanently submerged channels, as well as in a tributary stream (Mpophomeni) at False Bay (Tables 1–2, Appendix). Two of these freshwater species, i.e. *Aplexa marmorata* and *Tarebia granifera*, are actually alien invasives that have spread from colonies initially restricted to the seepage points around Catalina Bay (Appendix). *Tarebia granifera* was recorded in high abundance at Makakatana in March 2012, spreading subsequently throughout the Narrows to the south and at least as far as Charter’s Creek to the north-west (Figure 1, Table 1).

*Cassidula labrella*, *Cerithidea decollata*, *Melampus parvulus* and *Melampus semiaratus* are the only mangrove species that have been able to survive within the system, despite the closed mouth conditions that have prevailed since 2002. *Cerithidea decollata* was the only one among them to be found in abundance at all mangrove sites, including the St Lucia Bridge, Back Channel, Honeymoon Bend (Narrows) and Shark Basin near the St Lucia Mouth (Figure 1, Table 1). On the other hand, *Cassidula labrella*, *Melampus parvulus* and *Melampus semiaratus* were only found in the mangroves at Shark Basin and in very low numbers, on shaded mud surfaces and under fallen wood.

**Figure 3.**
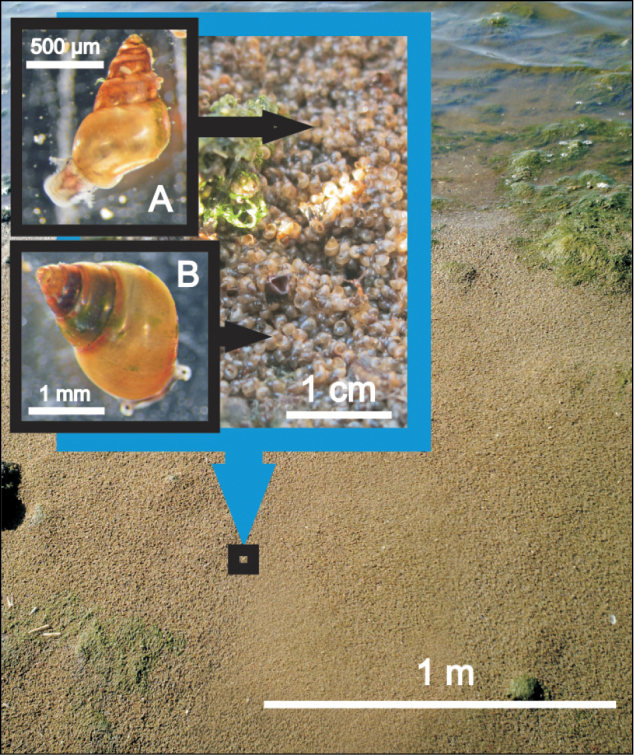
Assimineidae sp. (**A**) and *Assiminea* cf. *capensis* (**B**): thick layer of snails washed up on the shoreline of Lister’s Point at False Bay in July 2012 (Photo: Nelson AF Miranda).

Of the typical estuarine species recorded in all surveys undertaken in the past in Lake St Lucia, only *Assiminea* cf. *capensis* and Assimineidae sp. persisted through the latest survey (Miranda et al. 2014). Large aggregations of *Assiminea* cf. *capensis* and Assimineidae sp. were found in various areas of the estuarine system, with the former generally preferring salinities below 30 and the latter dominating under hypersaline conditions, mainly at Lister’s Point (False Bay). In July 2012, accumulations of hundreds of thousands of live individuals, mainly belonging to Assimineidae sp., were observed along the shoreline of Lister’s Point, apparently washed up by wind-driven wave motion (Figure 3). Similar aggregations, but this time dominated by *Assiminea* cf. *capensis*, were observed the following year, in May 2013, in the bay just south of Lister’s Point, following the onset of a wet cycle with flooding and consequent drop in salinity to about 15–20 throughout False Bay. The oceanic pelagic heteropod *Pterotrachea* cf. *hippocampus* was only recorded on one occasion in August 2013, while netting zooplankton in the beach spillway connecting the Mfolozi to the St Lucia Mouth.

**Figure 4.**
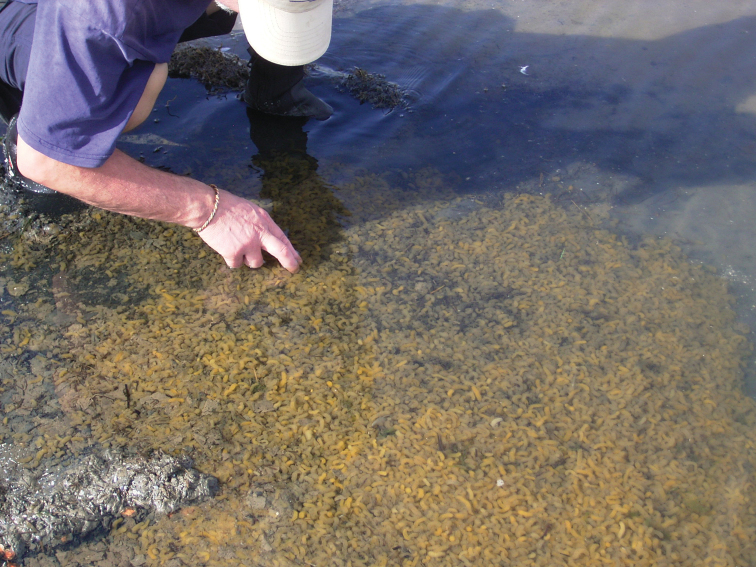
*Haminoea natalensis*: Aggregation of egg masses spawned during September 2006 in the shallows of Catalina Bay, on the Eastern Shores of South Lake (Photo: Lynette Clennell).

Species that were not found alive during the 2012–2013 survey, but have been previously documented as dominant within the system include *Nassarius kraussianus* and *Haminoea natalensis*. Both were only recorded as dead shells during 2012-2013, but in very large numbers and throughout the lake basins, particularly at Charter’s Creek, Catalina Bay and Fani’s Island (South Lake) (Figure 1, Table 1). While the last live record of *Nassarius kraussianus* during the recent closed mouth phase of the estuary dates back to the spring of 2006 (MacKay et al. 2010), *Haminoea natalensis* was found alive in large abundance at least until 2011. In the South Lake, at Charter’s Creek and Catalina Bay dense aggregations of freshly-spawned egg masses were observed in the spring of 2006 (Figure 4).

Among the mangrove dwellers that were present in the past but have recently disappeared entirely from the system are all the *Littoraria* species, i.e. *Littoraria coccinea glabrata*, *Littoraria intermedia*, *Littoraria pallescens*, *Littoraria subvittata* and *Littoraria scabra* (Table 2, Figure 2). Although the estuarine lake has experienced a large freshwater inflow since late 2011, several freshwater species that were previously found within the system were not recorded alive in 2012–2013. These include both *Neritina* species, i.e. *Neritina gagates* and *Neritina natalensis*, as well as *Bulinus tropicus*, *Bulinus forskalii*, *Pseudosuccinea columella* and *Oxyloma patentissima* (Figure 2, Appendix).

Typical estuarine and/or coastal marine species that are also among the new records may have entered the system only on sporadic occasions and/or for short periods of time under open mouth conditions. They include *Afrolittorina africana*, *Alaba pinnae*, *Ergalatax heptagonalis* and *Phalium areola*. All of them are represented in past collections from the KwaZulu-Natal Museum (Table 2). The common coffee-bean snail *Melampus lividus*, the estuarine limpet *Siphonaria oculus* and the opisthobranch *Bursatella leachii* were already reported in the earliest surveys of the University of Cape Town (Day et al. 1954; Millard and Broekhuysen 1970) (Table 2, Figure 2, Appendix). A second opisthobranch species, *Stylocheilus striatus*, was only recorded at Catalina Bay for a few months, immediately after the seaward mouth breach of March 2007. Finally, coastal marine species that were clearly once established in the northern lakes and have only been recorded in the latest survey from dead shells include whelks such as *Murex brevispinus* and *Purpura bufo*, and the cerithiid *Cerithium dialeucum* (Table 2, Appendix).

## Discussion

### Gastropod diversity and hydrological phases

Major climatic events and hydrodynamic processes control the gastropod species richness and abundance in the St Lucia estuarine lake (Figure 2). The highest diversity reported so far coincides with the period Jul 1964 – Jan 1965, when the second survey by the University of Cape Town (UCT) was conducted on the system. On that occasion, 12 gastropod species were recorded at a time when the estuarine lake was under tidal influence, with a normal salinity gradient decreasing from the estuary basin to the northern lakes (Millard and Broekhuysen 1970). Other major surveys undertaken by UCT in 1948-1951 and by NMSA in 1987 resulted in total records of 7 and 8 species, respectively (Day et al. 1954; Figure 2). These were periods characterized respectively by the first hypersaline event recorded in the system and a flood peak flow occurrence (Stretch et al. 2013).

During the last decade, St Lucia has undergone some of the most dramatic shifts ever recorded in the region. These have caused an unprecedented crisis and triggered a burst of fresh research activity on the system. It is thus not surprising that of the total 37 species of gastropod recorded within the estuarine lake, 19 are new records arising from the recent escalation in analyses and collecting efforts. During the latest dedicated gastropod survey, undertaken between Jan 2012 and Nov 2013, a total of 15 species were recorded, with only 12 found still alive and four in reasonable abundance, even if intermittently. Among the latter group, two are actually alien invasive species, i.e. *Tarebia granifera* and *Aplexa marmorata* (Figure 2).

### Response to recent dry and wet phases

In 2002, a sand berm closed off the St Lucia Estuary from the ocean, leading to a prolonged period of mouth closure, which still persists currently. The mouth was breached from the seaward side for a brief period of six months, between March and August 2007, by a combination of extreme events linked to Cyclone Gamede (Whitfield and Taylor 2009). Between 2002 and 2011, prolonged closure and low rainfall resulted in frequent periods of hypersaline conditions in the northern lakes, with complete desiccation of some areas at times (Perissinotto et al. 2013). These events led to the virtual disappearance of the entire gastropod community, with the exception of few mangrove dwellers and the most tolerant estuarine species, namely *Assiminea* cf. *capensis*, Assimineidae sp. and *Haminoea natalensis*. These were actually able to thrive, as little competition for resources remained at the onset of the harsh conditions (Figures 2–4). Alien invasive species, such as *Tarebia granifera* and *Aplexa marmorata*, were also able to take advantage of this situation and occupy vacant habitats and under-utilized resources in freshwater seepage areas.

Since the end of 2011, the system has entered a wet phase, with above average rainfall leading to occasional flooding and the prevalence of oligo- to polyhaline conditions throughout the extent of the system. This was compounded by the excavation of a beach spillway in July 2012, which has since contributed substantial freshwater inflow from the Mfolozi River into the St Lucia system and also partial exchange of water with the open ocean (Nel 2014, van Elden et al. in press). These changes have led to the appearance of a number of new gastropod species of brackish to freshwater origin, including *Bulinus natalensis*, *Cassidula labrella*, *Melampus parvulus*, *Melampus semiaratus* and *Melanoides tuberculata* (Figure 2). While the presence of the heteropod *Pterotrachea* cf. *hippocampus* in recent plankton collections clearly indicates marine penetration into the St Lucia, although the beach spillway connection to the Mfolozi mouth has failed so far to result in significant recruitment of typical coastal gastropod species from the ocean.

### Historical trends

Historical collections and surveys have, however, recorded numerous species of euryhaline marine and estuarine species, even in the uppermost reaches of the estuarine lake. For instance, the tick shell *Nassarius kraussianus* is present in all museum collections from St Lucia and has been recorded as abundant in all previous studies in the area (Table 2, Figure 2). Despite the numerous dead shells retrieved during the past few years throughout the system, it has not been found alive since 2006 (MacKay et al. 2010). *Nassarius kraussianus* is known as being generally associated with *Zostera* beds (Marais and Seccombe 2010) and its recent disappearance from the system has coincided with the extinction of the *Zostera* beds inside the lake basins after 2005, following the closure of the mouth in 2002 (Adams et al. 2013). Similarly dependent on marine grasses are typical estuarine species, such as *Alaba pinnae*, which is generally attached to *Zostera* blades (Kilburn and Rippey 1982), and the top-shell *Jujubinus suarezensis* and the whelk *Murex brevispinus*, both tidal mudflat dwellers living among submerged seagrasses (e.g. *Thalassodendron* in Mozambique, Kilburn and Rippey 1982). Only old specimens of these species were found reposited in the KwaZulu-Natal Museum (Table 2) and only dead shells were retrieved in the latest survey. Therefore, it seems most likely that these too may have died out shortly after the closure of the St Lucia mouth. Other mouth closure episodes have been recorded in the past, e.g. 1959-1961 and 1992-1993 (Figure 2), however they never persisted uninterruptedly for such a long period of time and never created conditions severe enough to cause the complete extinction of the *Zostera* beds from the system (Millard and Broekhuysen 1970; Adams et al. 2013).

Apart from causing the disappearance of marine grasses, prolonged mouth closure would also lead to the eventual death of barnacle and oyster beds (Nel et al. 2012), on which several species of gastropods depend for their food. For instance, the two whelks retrieved at False Bay and Charter’s Creek (South Lake) as dead shells in recent collections, *Ergalatax heptagonalis* and *Purpura bufo*, are known to be associated with barnacles and most probably depended on the dense barnacle beds that proliferated on the Cretaceous rock platforms prior to mouth closure (Kilburn and Rippey 1982; Marais and Seccombe 2010).

### Mangrove communities

Among the 20 species not previously recorded from the St Lucia estuarine lake are typical mangrove dwellers, such as *Littoraria intermedia*, *Littoraria pallescens*, *Littoraria subvittata*, *Littoraria scabra* and *Melampus parvulus*. St Lucia mangroves have undergone significant deterioration since the mouth closed in 2002, as persistent low salinity in the Narrows and near the mouth has favoured the development of reeds at the expense of mangrove vegetation (Adams et al. 2013). This has inevitably impacted on the once rich mangrove-dependent gastropod community (Day et al. 1954; Millard and Broekhyusen 1970). Indeed, of all the typical mangrove species reported here, only the resilient *Cerithidea decollata* was found in reasonable numbers during the latest survey. The other three species still present, *Melampus semiaratus*, *Melampus parvulus* and *Cassidula labrella*, occurred sporadically in very low numbers and only near the St Lucia Mouth. Surprisingly, the giant mangrove whelk, *Terebralia palustris* was never recorded at St Lucia, despite having been reported as well-established and common both to the south (e.g. Richards Bay, Durban Bay) and to the north (Mgobezeleni, Kosi Bay) of this system, at least in the past (Macnae 1963; Berjak et al. 2011).

### Key and indicator species

Population explosions of the bubble shell *Haminoea natalensis* with its distinct egg masses were recorded seasonally until 2009 (Figures 2 and 4). The observed trend of population explosions followed by high mortality and dwindling numbers is typical for opisthobranchs. Environmental conditions during the different seasons as well as the recruitment of *Haminoea natalensis*, are the most important factors driving its population biology (Malaquias and Sprung 2005; Miranda et al. 2011). *Haminoea natalensis* has not been observed in St Lucia again after 2009. However, when favorable higher salinity conditions return, the species will probably again be found along the shores in shallow water, feeding on microphytobenthic mats (Miranda and Perissinotto 2012).

The taxonomy of assimineids, or sentinel snails, is poorly understood and currently under revision in South Africa. In the St Lucia Estuary, there are inconsistencies in the literature in terms of what species of *Assiminea* occur in the system. This is not surprising given the morphological and ecological similarities as well as spatial overlap between different assimineids. Earlier literature refers to *Assiminea bifasciata* as the only species present in the system (Day et al. 1954; Millard and Broekhuysen 1970; Boltt 1975; Whitfield and Blaber 1978). Recent literature reports *Assiminea ovata* (Miranda et al. 2011, Carrasco et al. 2012, Daly et al. 2012) and *Assiminea globifera* (Taylor et al. 2006) and *Assiminea durbanensis* (Weerts 1993) have also been reported from St Lucia. Three species of assimineids are listed by MacKay et al. (2010) in St Lucia, while Pillay and Perissinotto (2008) and Owen et al. (2010) make reference to *Assiminea* sp.. Millard and Broekhuysen (1970) reported the occurrence of *Assiminea bifasciata* as well as *Syncera* sp. *Syncera* Gray, 1821 is *nomen nudum* and a synonym of *Assiminea* (Fukuda and Ponder 2003). The most recent genetic and morphological study conducted in the St Lucia Estuary has confirmed the existence of two species: *Assiminea* cf. *capensis* and Assimineidae sp. (“*Assiminea*” aff. *capensis* in Miranda et al. 2014). These two species exhibit patterns of spatial overlap that appear to vary depending on environmental parameters, particularly salinity. Assimineidae sp. is an assimineid in the broader sense, but belongs in an as yet unnamed genus and subfamily (Winston Ponder pers. comm.). Perhaps what Millard and Broekhuysen (1970) reported as *Syncera* sp. was in fact Assimineidae sp.. This false sentinel snail seems to prefer the more saline conditions in the northern parts of St Lucia and has been previously referred to as *Coriandria durbanensis* or *Assiminea durbanensis* (Raw et al. 2013; Weerts 1993). It is clear that *Assiminea* cf. *capensis* and Assimineidae sp. have been misidentified and confused in the past because of poor taxonomic knowledge and their morphological variability and similarities.

### Alien invasive species

Three of the five predominant alien invasive freshwater gastropods in South Africa have been recently recorded from St Lucia: *Aplexa marmorata*, *Pseudosuccinea columella* and *Tarebia granifera*. Previously, under hypersaline conditions these species were restricted to freshwater seepage areas on the Eastern Shores of the South Lake and along the Narrows (Miranda et al. 2010). However, the oligohaline conditions which currently persist following an increased volume of freshwater entering the system potentially favour the expansion of these species to new areas. This has already been observed with *Tarebia granifera*, which due to its unexpected high salinity tolerance (Miranda et al. 2010) has recently been recorded, albeit at low densities, from Charter’s Creek and Makakatana on the Western Shores of South Lake.

As the freshwater-dominated phase of St Lucia continues, the potential for alien invasive gastropods to enter and spread within the system increases. The consequences of these expansions vary depending on the species. *Pseudosuccinea columella* in South Africa is susceptible to the liver flukes (*Fasciola* spp.) which infect livestock, although it has not been confirmed as an intermediate host (Appleton 2003). *Aplexa marmorata* is widespread in KwaZulu-Natal, however its potential impacts are largely unknown. Similarly, although not reported from St Lucia, is *Physa acuta* (de Kock and Wolmarans 2007) and *Helisoma duryi* (Appleton 2003). The latter has however been recorded from artificial environments in the region. The greatest potential impact is that of *Tarebia granifera*, which displaces native gastropods and attains very large densities (Miranda et al. 2011; Raw et al. 2013). As *Tarebia granifera* is so successful, this species poses a threat to native malacofauna, such as the *Bulinus* species group, which would be expected to expand their range during a freshwater-dominated phase.

In addition to the threats from freshwater alien invasive species, estuaries are also threatened by the invasion of marine species from coastal sources (Nehring 2006). The majority of marine alien invasive species are introduced through ship fouling or ballast water (Picker and Griffiths 2011) and the proximity of an estuary to intensive international shipping increases its risk to introduced species (Nehring 2006). For St Lucia, the largest threat comes from the shipping activities at the industrial port of Richards Bay. Presently, there are no records of marine alien species in St Lucia due to the prolonged closure of the mouth to the Indian Ocean. The recent re-establishment of a connection through the beach spillway with the Mfolozi River may allow previously reported native marine species to re-enter the system, but also increase the risk of introduction of alien species. The continuous monitoring and assessment of potential impacts that these species may have on the ecosystem at large is necessary, given the significance of St Lucia as the largest estuarine lake in Africa and South Africa’s first World Heritage Site.

## Conclusions

Throughout its history, the St Lucia estuarine lake has experienced drastic shifts in hydrological states, from extreme dry conditions accompanied by hypersalinity and desiccation, to floods followed by freshwater dominance. The state of the mouth has also varied from an extended open bay joined to the Mfolozi River to extreme constriction and prolonged closure. The latest period of closure has been unprecedented and virtually uninterrupted since 2002. Although the monitoring of gastropod diversity within the system has been erratic until recently, there are clear indications that higher diversity has coincided with periods when the estuarine lake was under tidal influence, with a normal salinity gradient decreasing from the estuary basin to the northern lakes (e.g. 1964–1965). Drastic declines were observed when the system experienced hypersaline (e.g. 1948–1951) or flood conditions (e.g. 1987), with a closed mouth state compounding the problem by preventing any recruitment from the ocean. During the last decade, St Lucia has undergone some of the most dramatic shifts ever recorded in the region. Despite the intense, dedicated gastropod surveys undertaken in 2012-2013, only 12 species were found still alive, with four in reasonable abundance. Among these, unfortunately two are actually alien invasive species, i.e. *Tarebia granifera* and *Aplexa marmorata*, with the first spreading at alarming rates as low salinity conditions now prevail throughout the system.

## Figures and Tables

**Figure:**
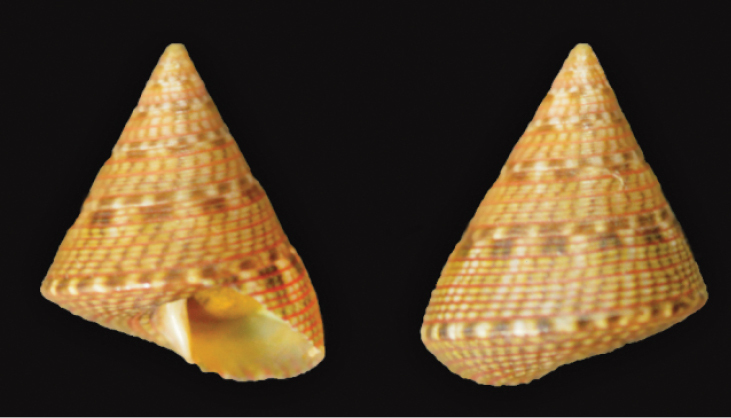
*Jujubinus suarezensis* (P. Fisher, 1978) 17 mm, Inhaca Island, Jul 1970 RN Kilburn leg.

**Figure:**
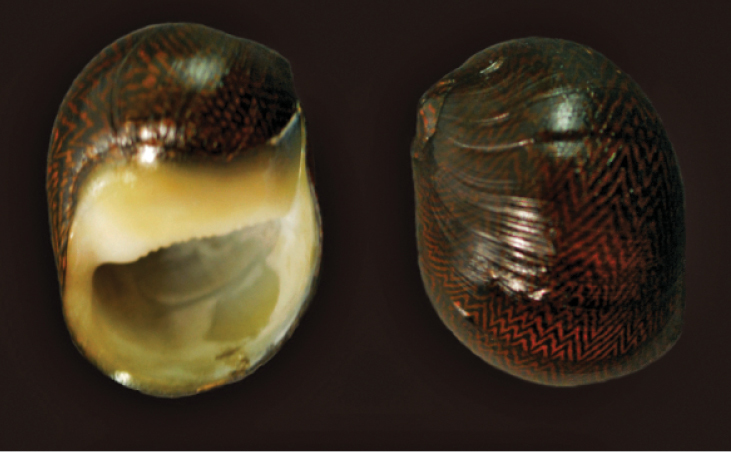
*Neritina gagates* Lamarck, 1822 19 mm, Mhlanga Estuary, Feb 2013 NAF Miranda leg.

**Figure:**
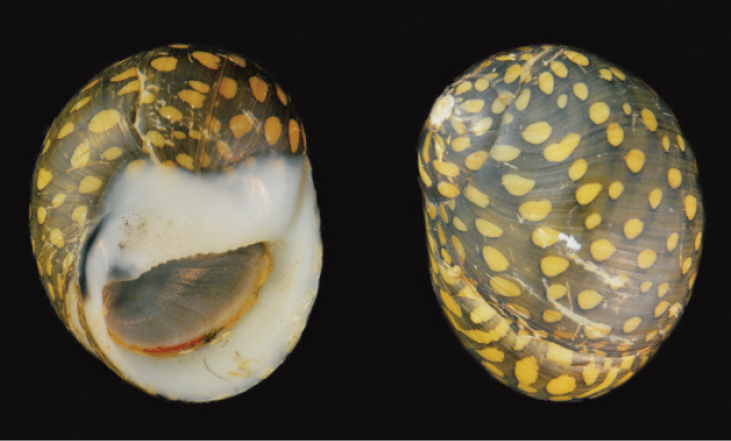
*Neritina natalensis* Reeve, 1855 23 mm, St Lucia False Bay, Oct 1924 HWB Marley leg.

**Figure:**
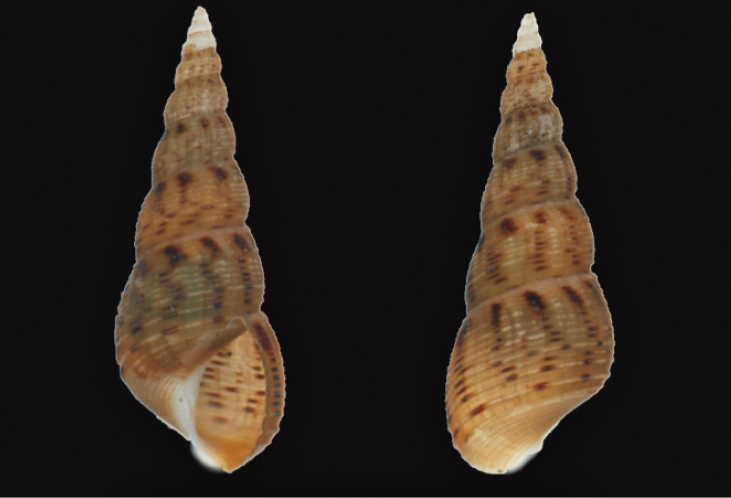
*Melanoides tuberculata* (Müller,1774) 23 mm, St Lucia False Bay, Feb 2013 NAF Miranda leg.

**Figure:**
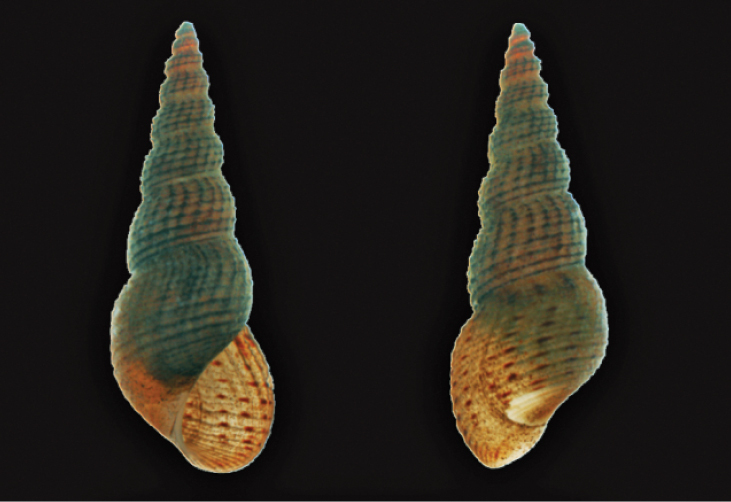
*Melanoides tuberculata* (Müller, 1774) 14 mm, St Lucia Mouth, Feb 2013 NAF Miranda leg.

**Figure:**
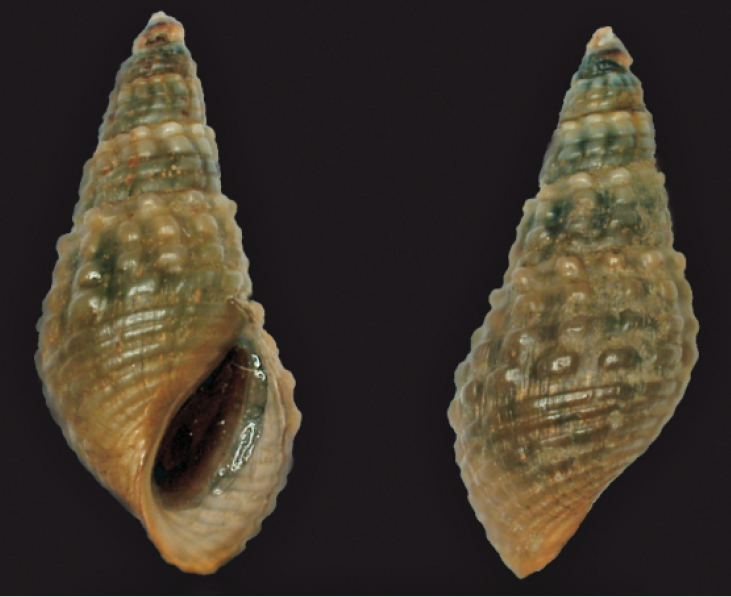
*Tarebia granifera* (Lamarck, 1822) 21 mm, St Lucia Catalina Bay, Feb 2010 NAF Miranda leg.

**Figure:**
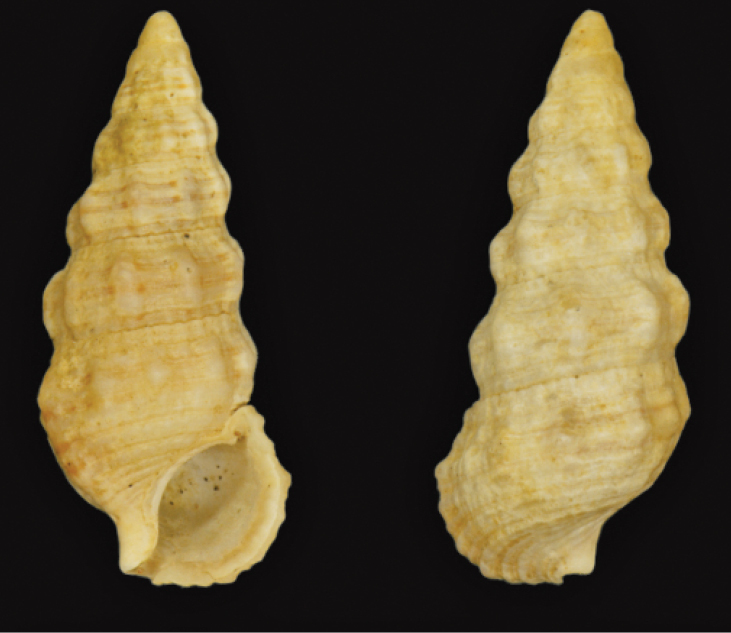
*Cerithium dialeucum* Philippi, 1849 30.5 mm, St Lucia False Bay, Mar 2012 R Perissinotto leg.

**Figure:**
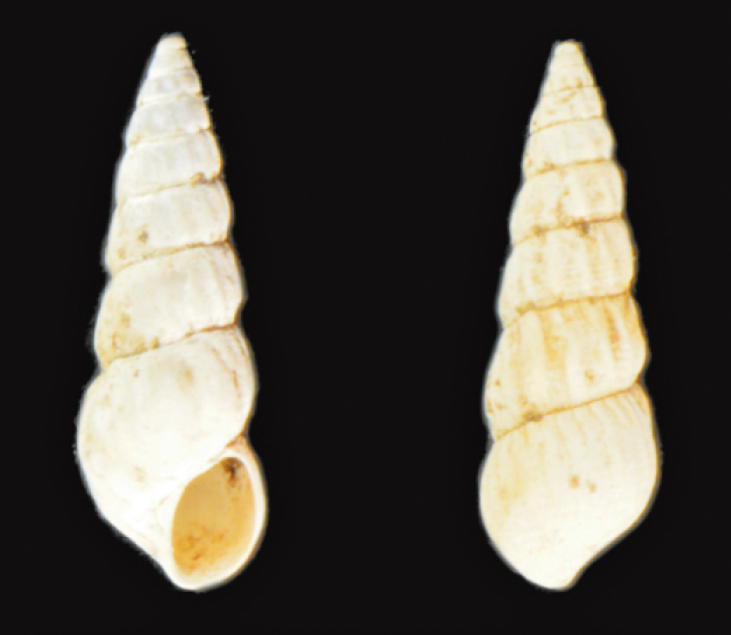
*Alaba pinnae* (Krauss, 1848) 9.3 mm, St Lucia Charter’s Creek,1967 R Kilburn leg.

**Figure:**
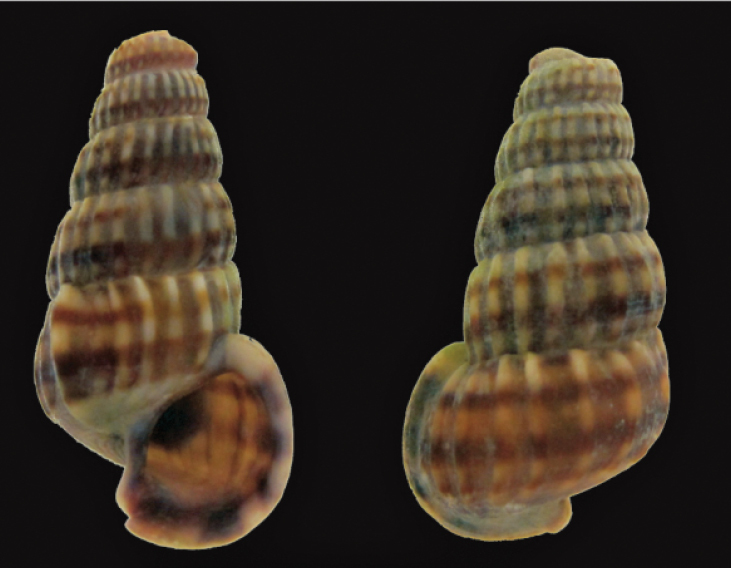
*Cerithidea decollata* (Linnaeus, 1767) 36 mm, St Lucia Bridge, Nov 2012 NAF Miranda leg.

**Figure:**
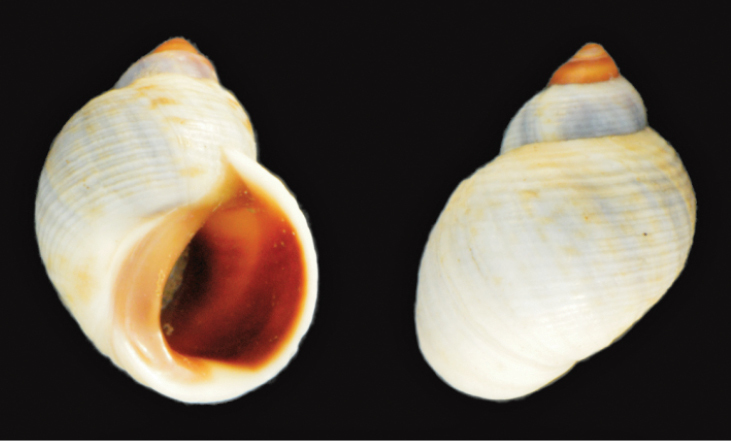
*Afrolittorina africana* (Krauss in Philippi, 1847) 7.8 mm, Zululand, Sep 1971 R Kilburn leg.

**Figure:**
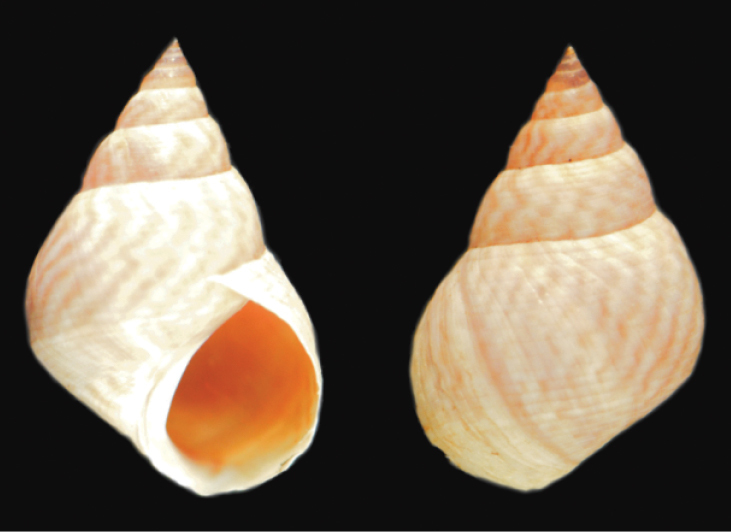
*Littoraria coccinea glabrata* (Philippi, 1846) 14 mm, St Lucia Estuary, Nov 1971 R Kilburn leg.

**Figure:**
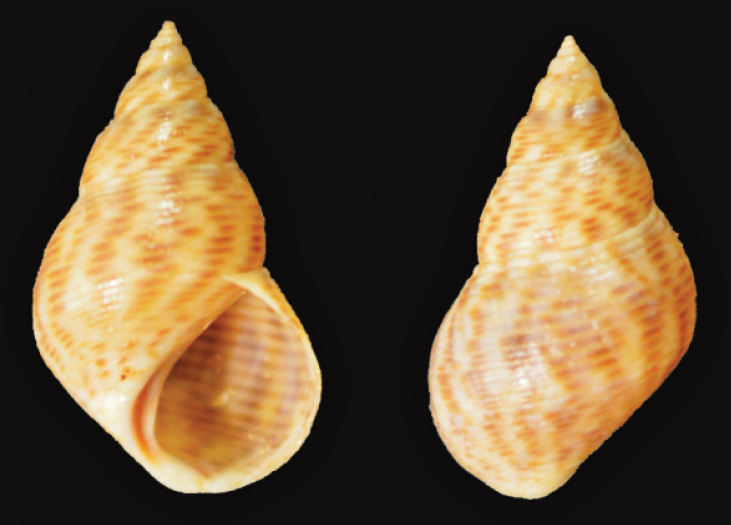
*Littoraria intermedia* (Philippi, 1846) 13 mm, St Lucia River Estuary, Nov 1971 RN Kilburn leg.

**Figure:**
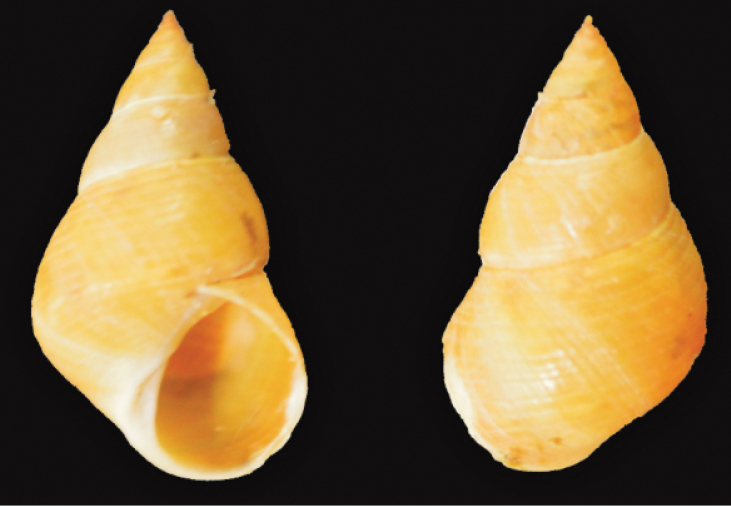
*Littoraria pallescens* (Philippi, 1846) 15 mm, St Lucia Estuary, Aug 1987 RH Taylor leg.

**Figure:**
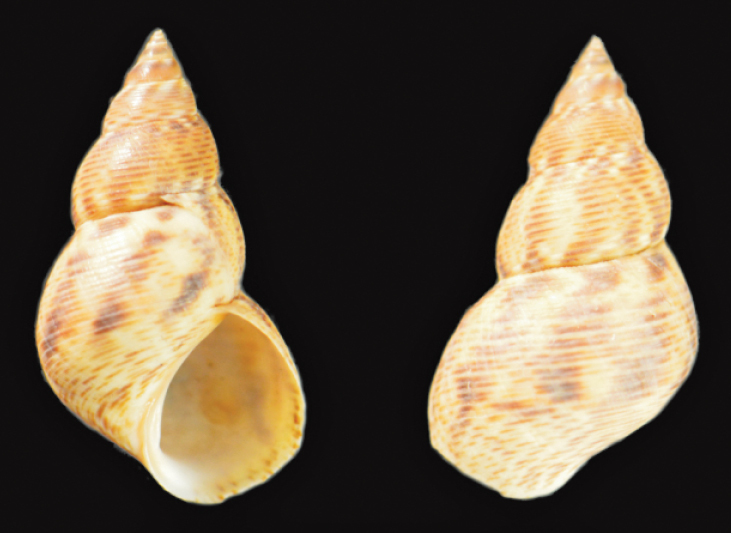
*Littoraria subvittata* (Reid, 1986) 18 mm, Lake St Lucia Burnup Collection.

**Figure:**
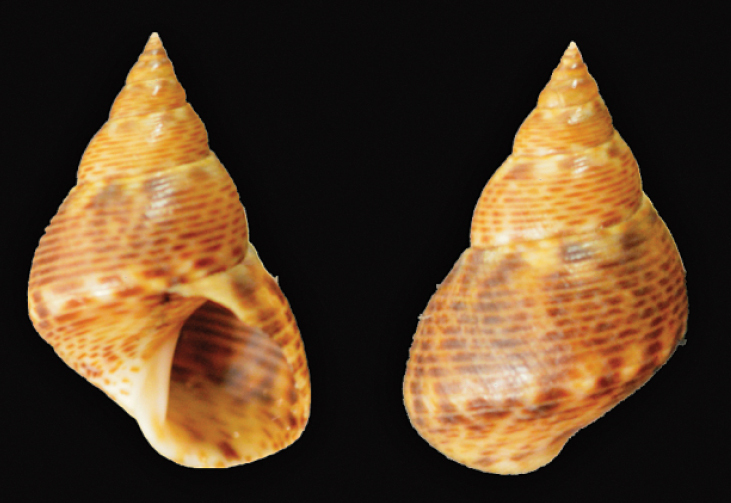
*Littoraria scabra* (Linnaeus, 1758) 14 mm, Kosi Bay Estuary, July 1987 DG Herbert leg.

**Figure:**
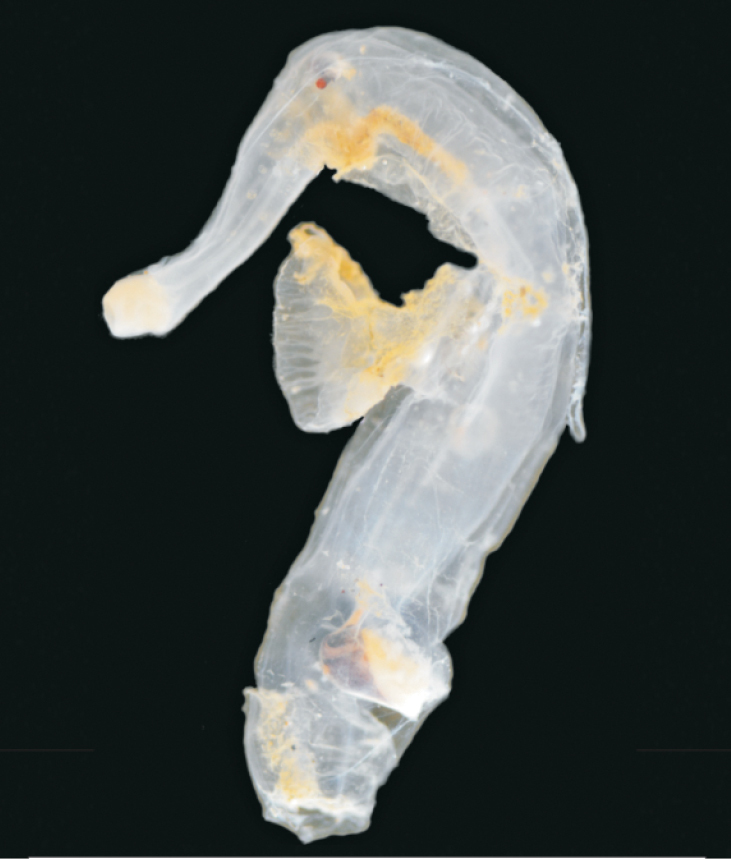
*Pterotrachea* cf. *hippocampus* Philippi, 1836 34 mm, Mfolozi Channel, Aug 2013 NK Carrasco leg.

**Figure:**
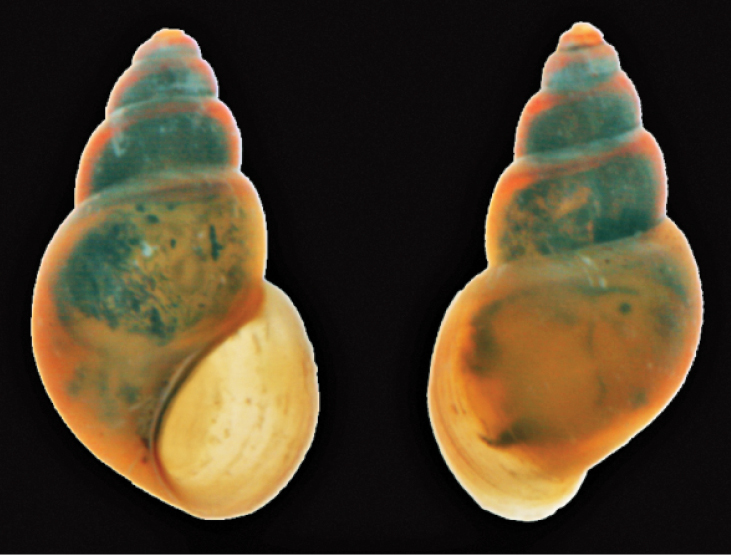
Assimineidae sp. 0.8 mm, St Lucia False Bay, Jul 2012 NAF Miranda leg.

**Figure:**
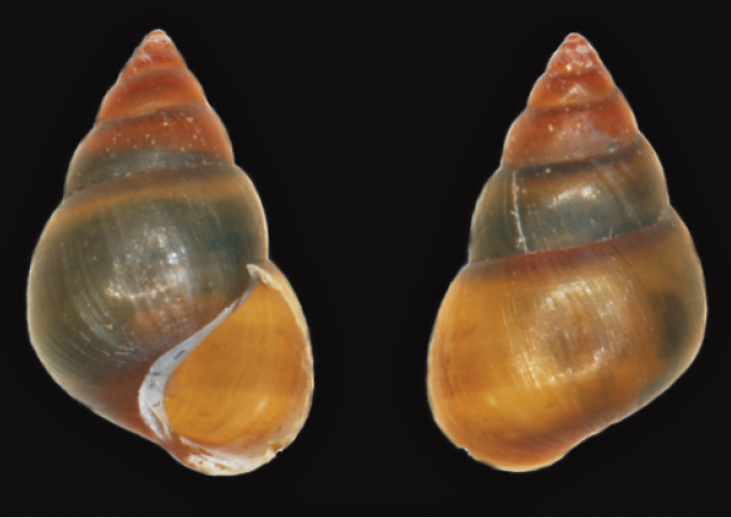
*Assiminea* cf. *capensis* Bartsch, 1915 2 mm, St Lucia Catalina Bay, Nov 2012 NAF Miranda leg.

**Figure:**
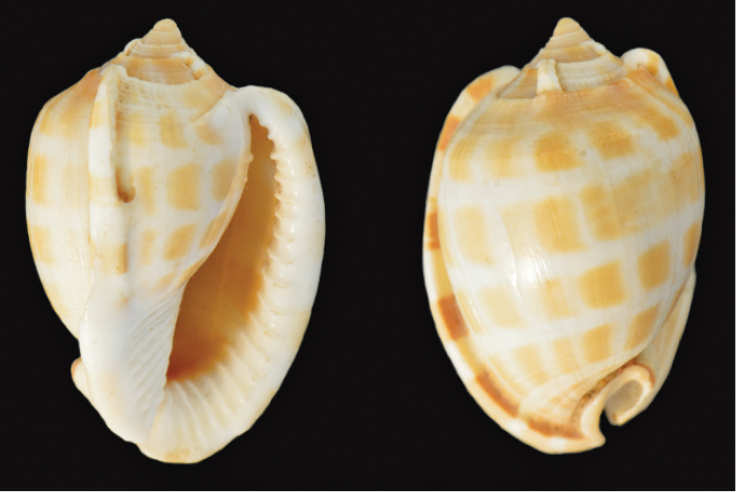
*Phalium areola* (Linnaeus, 1758) 55 mm, St Lucia Bay, 1978 M Lavoipierre leg.

**Figure:**
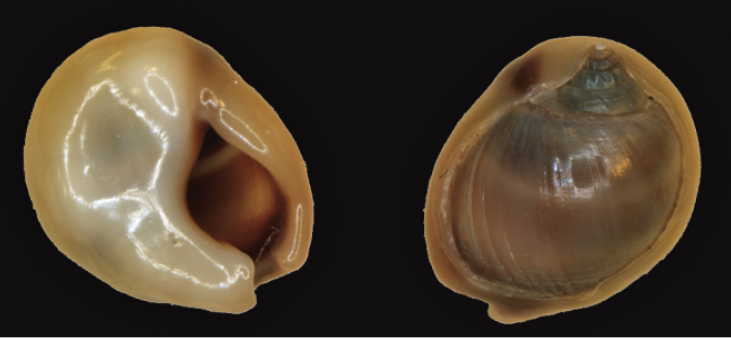
*Nassarius kraussianus* (Dunker, 1846) 11 mm, St Lucia Charter’s Creek, Mar 2012 NAF Miranda leg.

**Figure:**
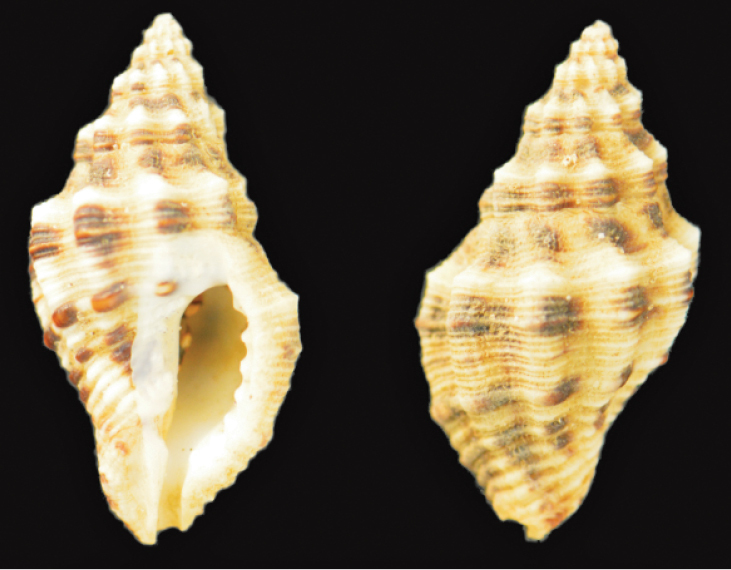
*Ergalatax heptagonalis* (Reeve, 1846) 25 mm, Mission Rocks, Apr 1987 R Kilburn leg.

**Figure:**
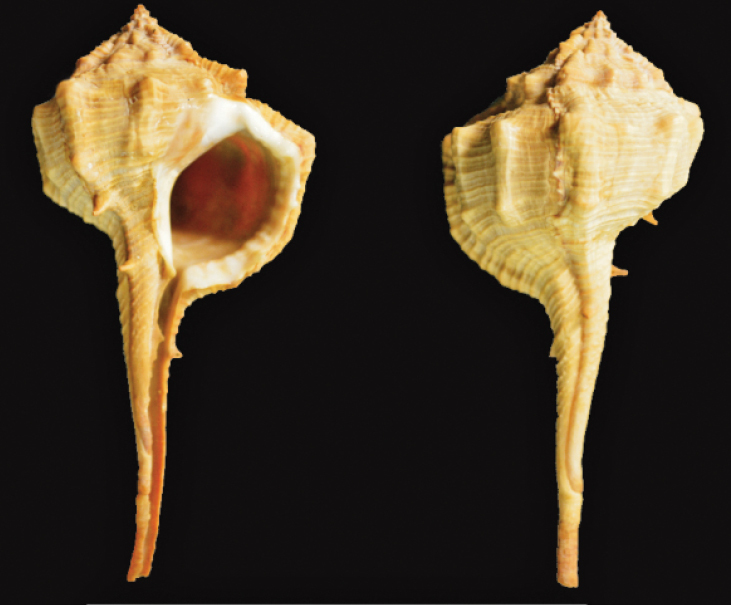
*Murex brevispinus* Lamarck, 1822 71 mm, Durban, Aug 1968 BJ Young leg.

**Figure:**
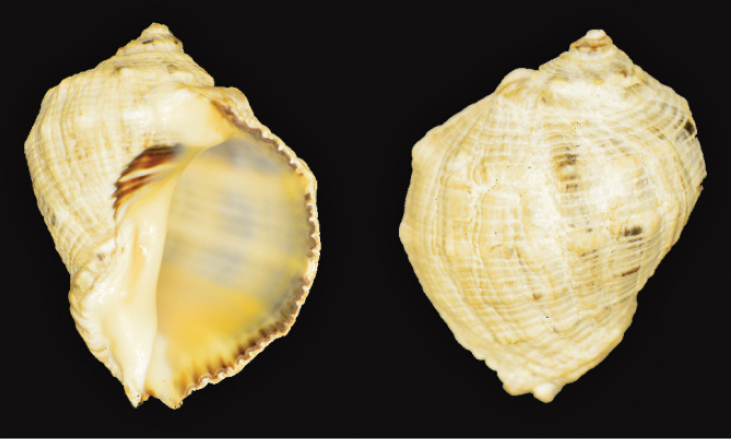
*Purpura bufo* Lamarck, 1822 52 mm, St Lucia False Bay, Jul 2013 NAF Miranda leg.

**Figure:**
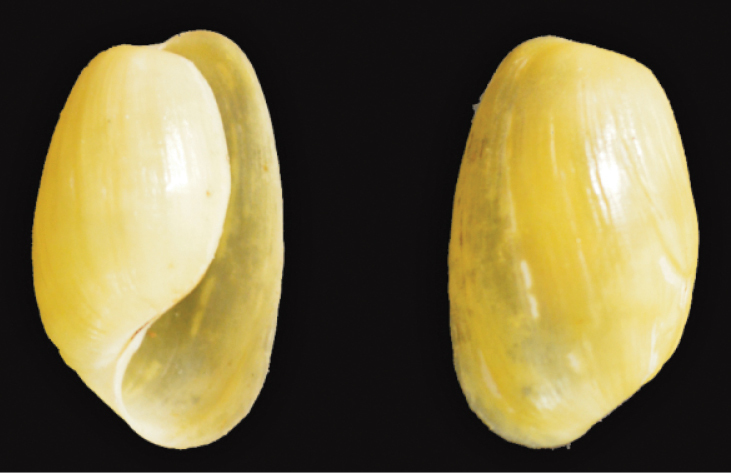
*Haminoea natalensis* (Krauss, 1848) 18 mm, St Lucia Charter’s Creek, Apr 1963 AC van Bruggen leg.

**Figure:**
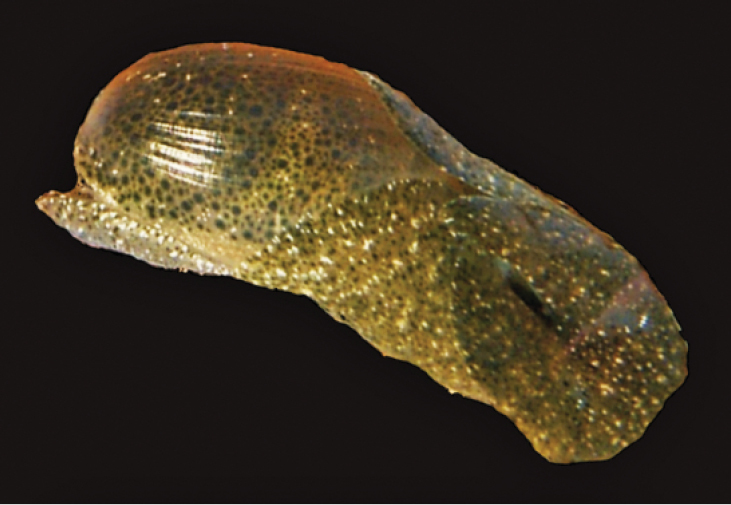
*Haminoea natalensis* (Krauss, 1848) 11 mm, St Lucia Catalina Bay, May 2007 L Clennell leg.

**Figure:**
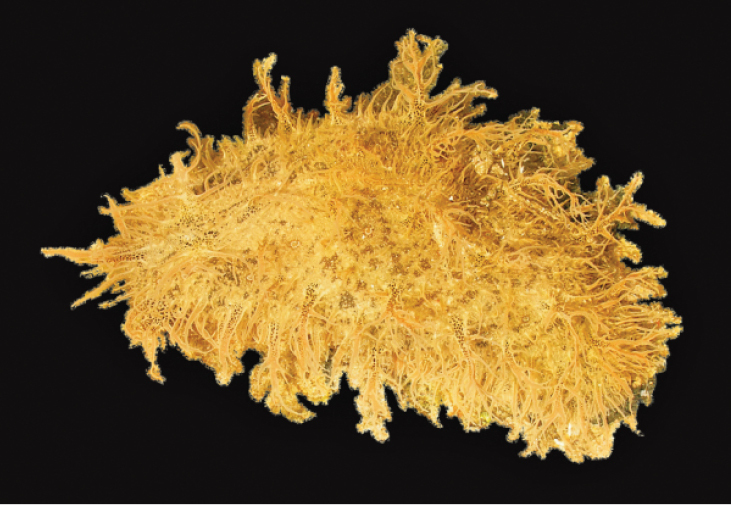
*Bursatella leachii* Blainville, 1817 175 mm, Salt Rock, Aug 2009 DG Herbert leg.

**Figure:**
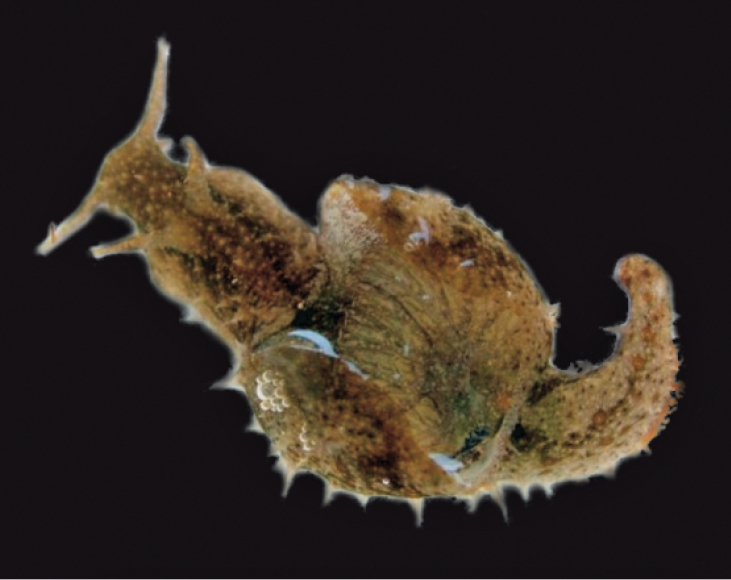
*Stylocheilus striatus* (Quoy & Gaimard, 1832) 53 mm, St Lucia Estuary, Apr 2007 C Fox leg.

**Figure:**
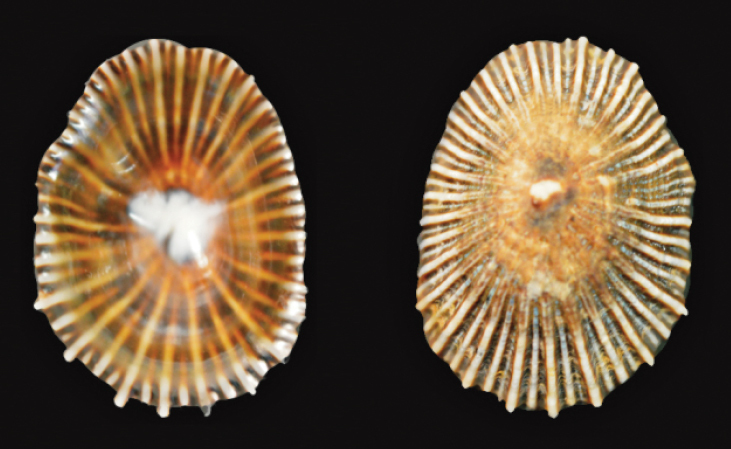
*Siphonaria oculus* Krauss, 1848 30 mm, Durban Bay, Sep 1972 BJ Young leg.

**Figure:**
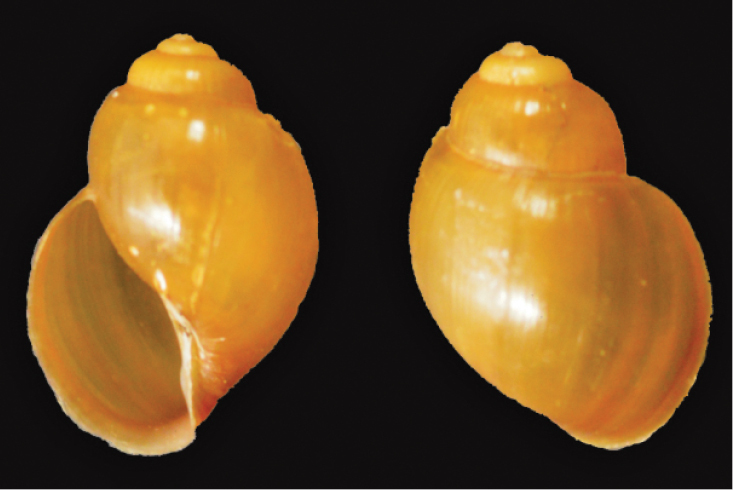
*Bulinus natalensis* (Krauss in Küster, 1841) 14 mm, St Lucia Catalina Bay, Nov 2012 JL Raw leg.

**Figure:**
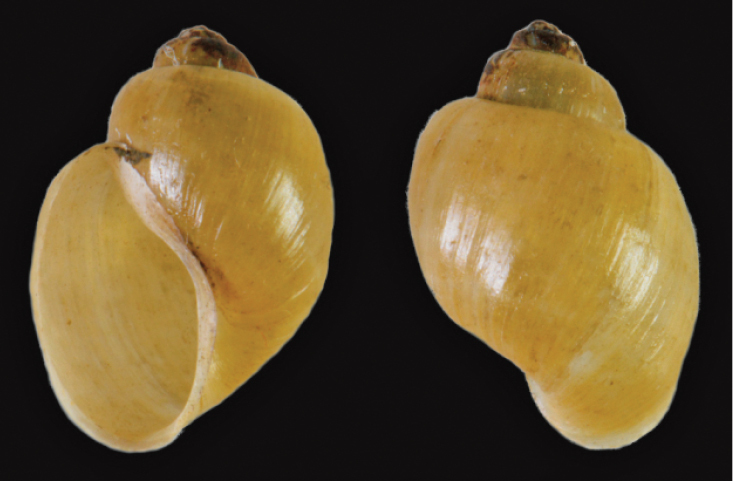
*Bulinus tropicus* (Krauss, 1848) 16 mm, Durban, Mar 2002 D Nadasan leg.

**Figure:**
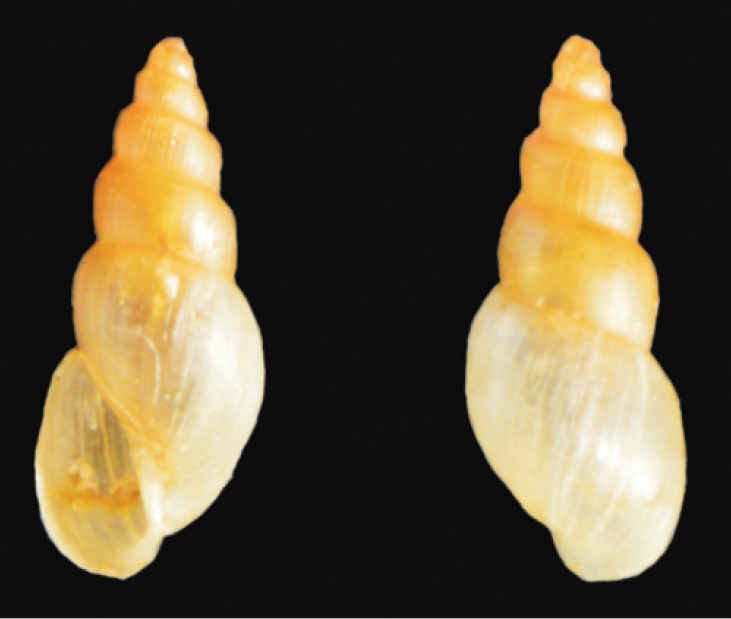
*Bulinus forskalii* (Ehrenberg,1831) 8.1 mm, Uvongo, Mar 1996 M Coke leg.

**Figure:**
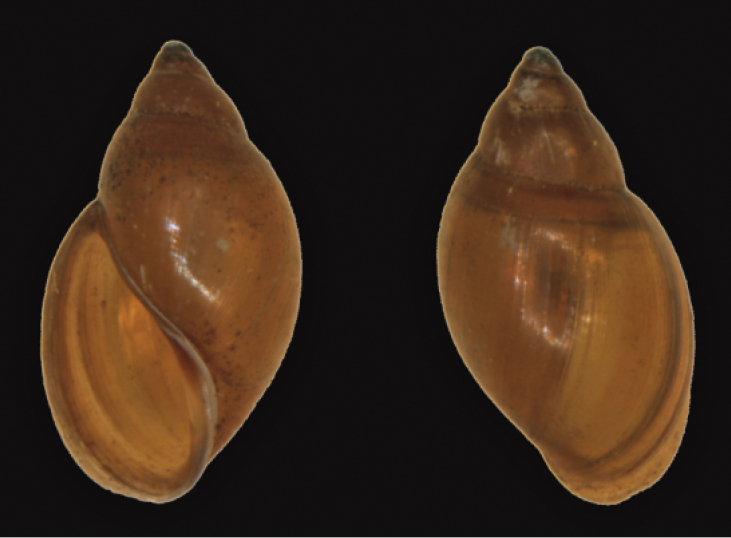
*Aplexa marmorata* (Guilding, 1828) 12 mm, St Lucia Catalina Bay, Feb 2010 NAF Miranda leg.

**Figure:**
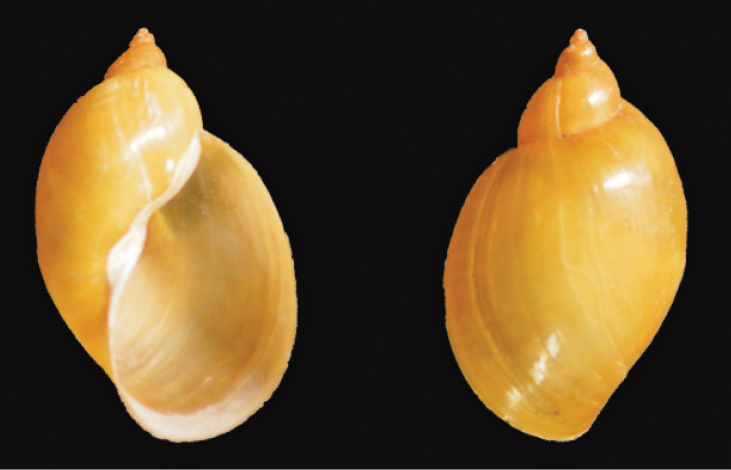
*Lymnaea natalensis* (Krauss, 1848) 12 mm, St Lucia Catalina Bay, Nov 2012 NAF Miranda leg.

**Figure:**
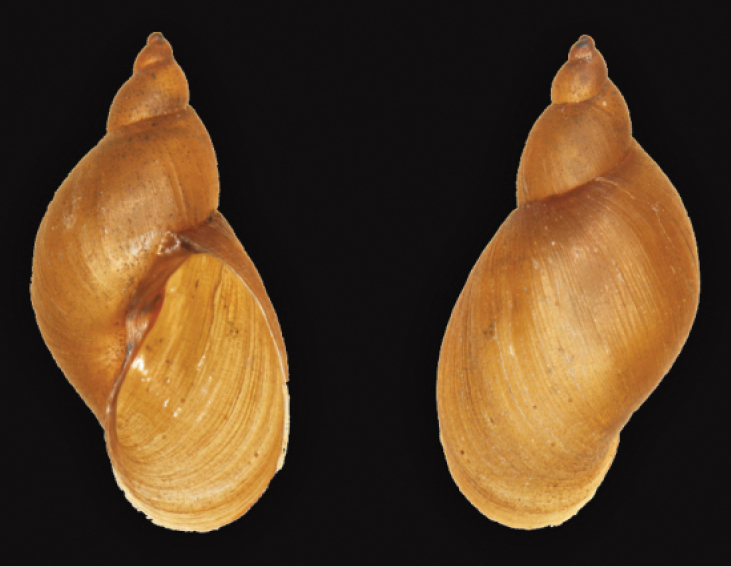
*Pseudosuccinea columella* (Say, 1817) 11 mm, St Lucia Catalina Bay, Jul 2009 NAF Miranda leg.

**Figure:**
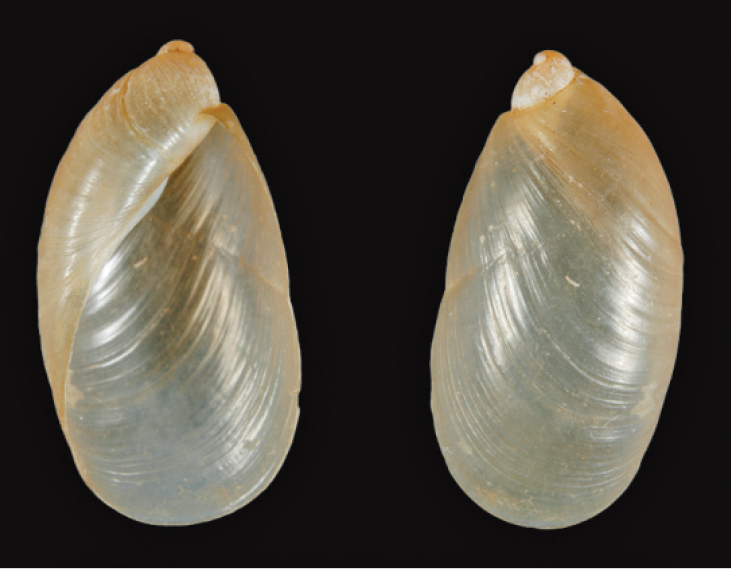
*Oxyloma patentissima* (Menke in Pfeiffer, 1853) 8.8 mm, Mhlanga Lagoon, Dec 1995 D Herbert & L Davis leg.

**Figure:**
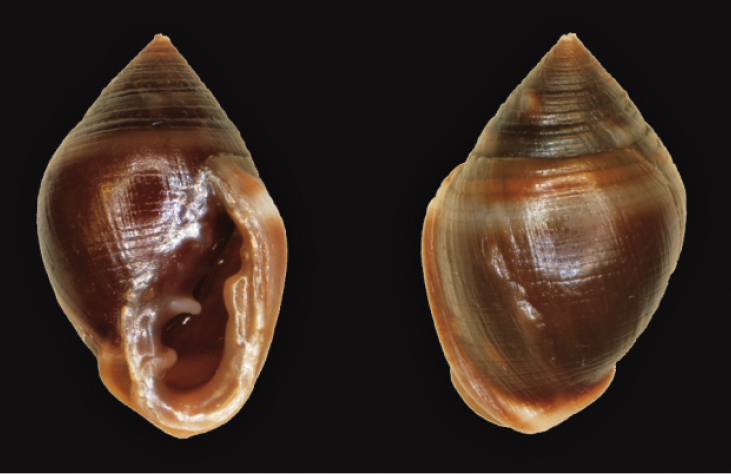
*Cassidula labrella* (Deshayes, 1830) 12 mm, St Lucia Mouth, Mar 2012 N Peer leg.

**Figure:**
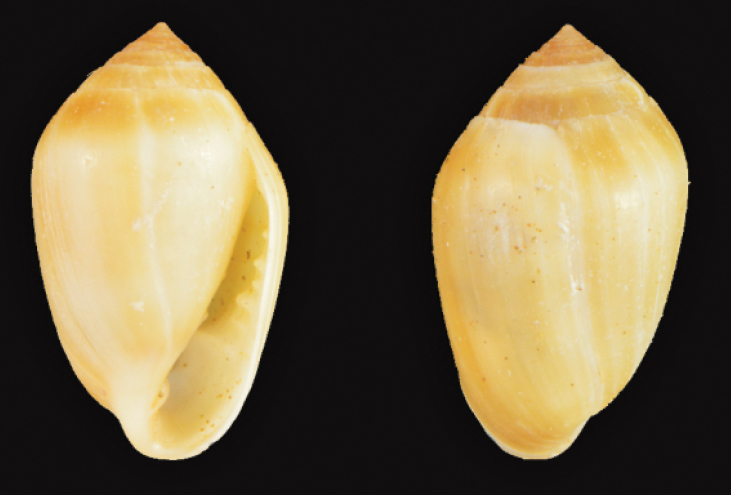
*Melampus lividus* (Deshayes, 1830) 18 mm, St Lucia Mission Rocks, Apr 1988 D Brink leg.

**Figure:**
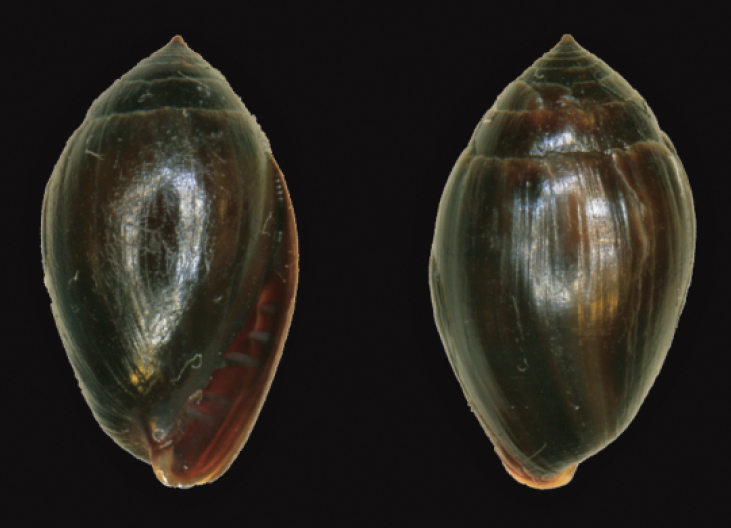
*Melampus parvulus* Pfeiffer, 1856 10 mm, St Lucia Mouth, Mar 2013 SM Ngubane leg.

**Figure:**
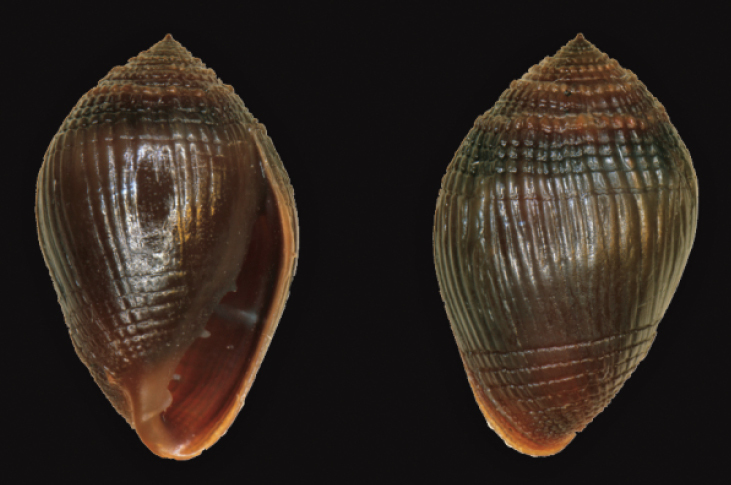
*Melampus semiaratus* Connolly, 1912 12 mm, St Lucia Mouth, Mar 2013 SM Ngubane leg.

## Appendix

Annotated and Illustrated Checklist of the Gastropod Molluscs of Lake St Lucia

### Family: Trochidae

#### *Jujubinus suarezensis* (P. Fischer, 1878)

**Common name.** Square top –snail.

**Size.** Maximum shell height 12 mm^(1)^.

**Remarks.** Occurs on protected mudflats^(1)^.

**Distribution.** East African distribution including Madagascar. Common in Mozambique extending south to Durban in South Africa^(1)^.

**St Lucia records.** Not previously reported from St Lucia; collected in 1987 (NMSA). Only dead shells collected in 2012 from Fani’s Island (South Lake).

### Family: Neritidae

#### *Neritina gagates* Lamarck, 1822

**Common name.** Brown nerite or Zebra nerite.

**Size.** Maximum shell height 22 mm^(2)^.

**Remarks.** Occurs on stones in streams that are tidally influenced^(2)^.

**Distribution.** Southeastern coast of Africa from Mozambique to Mzamba in the Eastern Cape Province of South Africa^(3)^. Also reported from the Comoro Islands, Madagascar and Seychelles^(2)^.

**St Lucia records.** Not previously reported from St Lucia; collected in 1988 from the St Lucia Mouth (NPB). Not found during the recent survey.

#### *Neritina natalensis* Reeve, 1855

**Common name.** Spotted nerite.

**Size.** Maximum shell height 20 mm^(2)^.

**Remarks.** Reportedly occurs within mangroves^(2)^ and on *Phragmites* stems in estuaries.

**Distribution.** East African coast from Somalia extending south to the Umzimkulu River on the KwaZulu-Natal coast of South Africa^(2)^. Also reported from the Mtamvuna River^(3)^.

**St Lucia records.** Not previously reported from St Lucia; collected in 1924 and 1987 (NMSA) from False Bay. Not recorded in the recent survey.

### Family: Thiaridae

#### *Melanoides tuberculata* (Müller, 1774)

**Common name.** Red-rimmed melania.

**Size.** Maximum shell height 45 mm^(3)^.

**Remarks.** Predominantly parthenogenetic species^(3)^ which inhabits fresh and brackish water^(2)^.

**Distribution.** Natural Indo-Pacific distribution includes much of Africa with a southern limit at Port Elizabeth^(2)^ and extending east to southern Asia and northern Australia^(3)^. Introduced to regions of North America^(4)^ and New Zealand^(5)^.

**St Lucia records.** Reported in 2012^(6)^ from Shark Basin (St Lucia Mouth). Recently recorded from the Mpophomeni Stream (False Bay).

#### *Tarebia granifera* (Lamarck, 1822)

**Common name.** Quilted melania^(7)^.

**Size.** Maximum shell height 29.5 mm^(7)^.

**Remarks.** Alien invasive species first recorded in northern KwaZulu-Natal in 1999^(8)^. Indigenous to southeastern Asia^(7)^ but has also been introduced to the Caribbean^(9)^, the Americas^(4,10)^ and Israel^(11)^.

**St Lucia records.** Reported from St Lucia in 2006^(12)^, 2007^(13)^ and between 2010 and 2013^(6,13,14)^. Reported in the recent survey from the mangroves at the St Lucia Bridge (Narrows) as well as Makakatana, Catalina Bay and Charter’s Creek (South Lake).

### Family: Cerithiidae

#### *Cerithium dialeucum* Philippi, 1849

**Common name.** White-studded cerith.

**Size.** Up to 30.5 mm.

**Remarks.** Dead shells collected at Lister’s Point in March 2012.

**Distribution.** Indo-Pacific distribution with southwestern reports from Mozambique^(15)^, Madagascar^(16)^ and Mauritius^(17)^. South African distrubution restricted to coastline from Kosi Bay to Durban^(18)^.

**St Lucia records.** Not previously reported.

### Family: Litiopidae

#### *Alaba pinnae* (Krauss, 1848)

**Common name.** Pinnated litiopid.

**Size.** Maximum shell height 11 mm^(1)^.

**Remarks.** Common estuarine species which varies in plumpness^(1)^.

**Distribution.** Still Bay to Zululand coast^(1)^.

**St Lucia records.** Not previously reported from St Lucia; collected at Charter’s Creek (South Lake) in 1967 and 1987 respectively (NMSA). Not found in the recent survey.

### Family: Potamididae

#### *Cerithidea decollata* (Linnaeus, 1767)

**Common name.** Truncated mangrove snail^(19)^.

**Size.** Maximum shell height 36 mm^(1)^.

**Remarks.** Common mangrove climbing whelk also found in salt marshes on mud beneath vegetation^(1)^, although less abundant^(19)^. Shells tend to have a characteristically decollated apex.

**Distribution.** Indo-pacific mangrove species. South African distribution extends from Knysna along southeastern coast into Mozambique^(19)^.

**St Lucia records.** Reported in previous surveys from 1948–51^(20)^, 1964–65^(21)^. Found in the recent survey at Shark Basin, at the Mfolozi Back Channel (St Lucia Mouth), and at the St Lucia Bridge (Narrows).

### Family: Littorinidae

#### *Afrolittorina africana* (Krauss in Philippi, 1847)

**Common name.** African periwinkle^(19)^.

**Size.** Shell height 5–8 mm^(19)^.

**Remarks.** Typical high intertidal species which occurs in large numbers usually on exposed rocks^(1)^.

**Distribution.** Southwestern Indian Ocean^(22)^ from Durban to southern Mozambique^(19)^ as well as southeastern Madagascar^(22)^.

**St Lucia records.** Not previously reported from St Lucia; collected in 1971 from “St Lucia River Estuary” (NMSA). Not recorded in the recent survey.

#### *Littoraria coccinea glabrata* (Philippi, 1846)

**St Lucia synonyms.** *Littorina glabrata* Philippi, 1846.

**Common name.** Striped periwinkle^(19)^.

**Size.** Maximum shell height 24 mm^(1)^.

**Remarks.** High resistance to desiccation^(19)^ enables habitation of exposed regions^(1)^ above high spring-tide level in sub-tropical regions.

**Distribution.** Tropical and subtropical Indian Ocean distribution^(1)^ with a southern limit at Port Elizabeth^(1)^ on the South African coast.

**St Lucia records.** Not previously reported from St Lucia; collected in 1971 (NMSA) from the St Lucia Estuary. Not found during the recent survey.

#### *Littoraria intermedia* (Philippi, 1846)

**Common name.** Estuarine periwinkle^(19)^.

**Size.** Shell height 14–26 mm^(23)^.

**Remarks.** Associated with roots and trunks of *Rhizophora* and occasionally *Bruguiera* mangrove trees^(23)^.

**Distribution.** Tropical and subtropical Indo-Pacific distribution from the east African coast including the Red Sea and east to Polynesia^(23)^, with a southern limit at Port Elizabeth on the Eastern Cape coast of South Africa^(19)^.

**St Lucia records.** Not previously reported from St Lucia; collected in 1971 and 1987 (NMSA) from St Lucia Estuary and Shark Basin respectively. Only dead shells retrieved recently from Shark Basin (St Lucia Mouth).

#### *Littoraria pallescens* (Philippi, 1846)

**Common name.** Pale periwinkle

**Size.** Shell height 15–25 mm^(23)^.

**Remarks.** Commonly occurs on the leaves of *Rhizophora* in mangrove forests^(23)^.

**Distribution.** Tropical to sub-tropical Indo-Pacific distribution extending from east African coast to Samoa^(23)^.

**St Lucia records.** Not previously reported from St Lucia; collected in 1987 (NMSA) from the St Lucia Estuary. Not found during the recent survey.

#### *Littoraria subvittata* (Reid, 1986)

**Common name.** Aldabra periwinkle.

**Size.** Shell height 13–34 mm^(23)^.

**Remarks.** Occurs in mangroves and marsh grass habitats as well as on sheltered rocks^(23)^.

**Distribution.** Coastal Indian Ocean distribution from Aden, Yemen^(23)^, in the north to the Swartkops River mouth^(24)^ at Port Elizabeth, South Africa. Range extends east to Mauritius, Maldives and Cocos Islands^(23)^.

**St Lucia records.** Not previously reported from St Lucia; collected by NMSA at unknown date. Not found during the recent survey.

#### *Littoraria scabra* (Linnaeus, 1758)

**Common name.** Mangrove periwinkle.

**Size.** Shell height 25–35 mm^(23)^.

**Remarks.** Characteristic mangrove and estuarine species found above high water^(1)^. Usually found on trunks, roots^(23)^ and branches of mangrove trees^(1)^.

**Distribution.** Tropical and sub-tropical Indo-Pacific distribution extending from the eastern African coast across to Samoa and Hawaii^(23)^. The southern limit of the species is Algoa Bay at Port Elizabeth in South Africa^(1,19)^.

**St Lucia records.** Reported from St Lucia in 1948–51^(20)^, 1964–55^(21)^ and 1972–73^(25)^ however the species has not been recorded recently.

### Family: Pterotracheidae

#### *Pterotrachea* cf. *hippocampus* Philippi, 1836

**Common name.** Sea elephant.

**Size.** Up to 34 mm.

**Remarks.** Pelagic marine species related to *Charonia* spp. (Ranellidae). The species is expected to occur more frequently in the system as the marine connection is maintained.

**Distribution.** Circumtropical distribution including the Gulf of Mexico^(26)^, the Mediterranean Sea^(27)^ and the North Atlantic^(28)^.

**St Lucia records.** Not previously reported from St Lucia; recorded in the recent survey in 2013 from the Mfolozi-St Lucia Beach Spillway (St Lucia Mouth).

### Family: Assimineidae

#### Assimineidae sp.

**St Lucia synonyms.** *Assiminea bifasciata* Nevill, 1880; *Coriandria durbanensis* (Tomlin, 1916); *Rissoa capensis* Sowerby, 1892.

**Common name.** False sentinel.

**Size.** Up to 2.22 mm.

**Remarks.** Taxon under revision. Provisional name based on genetics and shell morphometrics^(29)^. Small size and sympatric occurrence with *Assiminea* spp. caused previous confusion and misidentification. Forms dense aggregations on firm mud. **Distribution.** East coast of southern Africa.

**St Lucia records.** Probably combined in the 1948–51^(4)^ survey with *Assiminea* spp. Reported in 2012 as *Coriandria durbanensis* (Tomlin 1916)^(6)^ from Lister’s Point (False Bay). Found in the recent survey from Charter’s Creek (South Lake), Hell’s Gate (North Lake) and Lister’s Point (False Bay).

#### *Assiminea* cf. *capensis* Bartsch, 1915

**St Lucia synonyms.** *Assiminea bifasciata* Nevill, 1880; *Assiminea* cf. *ovata* (Krauss, 1848); *Syncera* sp. Gray, 1821;

**Common name.** Sentinel snail.

**Size.** Maximum shell height 7 mm^(1,19)^.

**Remarks.** Dominant in estuaries on the east coast^(19)^ forming large colonies on firm mud sheltered by mangroves or salt marsh^(19)^. Species listed as “*capensis*” following the recommendation of W. Ponder, as the taxonomy of this group is currently under review.

**Distribution.** Southern African species extending from Langebaan to Mozambique^(19)^.

**St Lucia records.** Previous records in 1948–51^(20)^, 1981–2^(30)^ and 2005^(12)^ probably comprised both *Assiminea* cf. *capensis* and “*Assiminea*” aff. *capensis*. Reported as *Syncera* sp. in 1964–5^(21)^ and *Assiminea durbanensis* in 1993^(31)^. Identified as *Assiminea* cf. *ovata* in 2007–9^(14)^ following Appleton 1996^(3)^. Reported as *Assiminea* cf. *capensis* in 2012^(6)^ and found during the recent survey from the Mfolozi Back Channel (St Lucia Mouth), Catalina Bay and Charter’s Creek (South Lake) as well as Lister’s Point (False Bay).

### Family: Cassidae

#### *Phalium areola* (Linnaeus, 1758)

**Common name.** Checkerboard bonnet.

**Size.** Maximum shell height 69 mm^(1)^.

**Remarks.** Inhabits sandy environments, known to bury itself in the substrate^(1)^. Subtidal species which was most likely washed into the system.

**Distribution.** Indo-Pacific distribution including the KwaZulu-Natal coast^(1)^. Reported from Mozambique^(15)^ and Tanzania^(32)^.

**St Lucia records.** Not previously reported from St Lucia; single specimen collected from the St Lucia Mouth by NMSA at an unspecified date. Not reported in the recent survey.

### Family: Nassariidae

#### *Nassarius kraussianus* (Dunker, 1846)

**St Lucia synonyms.** *Nassa kraussiana* (Dunker, 1846).

**Common name.** Tick shell^(19)^.

**Size.** Shell height 7.5–10 mm^(1)^.

**Remarks.** Characteristic estuarine species found in salt marshes and lagoonal mudbanks^(19)^ where it forms dense colonies. Occurs within shallow pools often among eelgrass^(1)^.

**Distribution.** Southern African distribution from the Namaqualand coast^(2)^ extending along KwaZulu-Natal^(19)^. Also reported from southern Mozambique^(15)^.

**St Lucia records.** Reported in 1948–51^(20)^,1972–73^(25)^ and 2010^(33)^. Recorded in 1964–65^(21)^ as *Nassa kraussiana*. Dead shells retrieved during the recent survey from South and North Lake shores, as well as False Bay.

### Family: Muricidae

#### *Ergalatax heptagonalis* (Reeve, 1846)

**Common name.** Heptagonal rock snail.

**Size.** Maximum shell length 30 mm^(1)^.

**Remarks.** Common under rocks or logs on muddy sand of estuarine bays^(1)^.

**Distribution.** Indo-pacific distribution including the KwaZulu-Natal coast^(1)^ extending to Mozambique as well as Madagascar^(15)^.

**St Lucia records.** Not previously reported from St Lucia; collected in 1987(NMSA) from the Mfolozi Link Canal (St Lucia Mouth). The specimen collected was probably exposed after excavation of the Link Canal as it is a Pleistocene subfossil. Not found during the recent survey, however the species may re-colonize St Lucia if the marine connection is maintained.

#### *Murex brevispinus* Lamarck, 1822

**Common name.** Short spined murex^(19)^.

**Size.** Maximum shell height 82 mm^(1)^.

**Remarks.** Occurs on protected intertidal sandbanks, often among eelgrass^(1,19)^. Forms dense mating aggregations^(19)^.

**Distribution.** Indian Ocean distribution along the eastern coast of Africa extending from Kenya to Durban^(1,15,19)^.

**St Lucia records.** Not previously reported from St Lucia; only dead shells retrieved from Lister’s Point (False Bay) and Fani’s Island (South Lake) in the recent survey.

#### *Purpura bufo* Lamarck, 1822

**Common name.** Toad purpura.

**Size.** Maximum shell height 70 mm.

**Remarks.** Common in rock pools on the KwaZulu-Natal coast. Specimen illustrated is a sub-adult.

**Distribution.** Tropical and sub-tropical Indian Ocean^(15,32)^ with southern limit in the eastern Transkei^(1,19)^.

**St Lucia records.** Not previously collected or reported from St Lucia. Only one dead shell was found at False Bay during the recent survey.

### Family: Haminoeidae

#### *Haminoea natalensis* (Krauss, 1848)

**St Lucia synonyms.** *Haminoea petersi* (Martens, 1879); *Haminoea gracilis* (Sowerby, 1897); *Cylichna africana* Bartsch, 1915; *Cylichna tubulosa* Gould, 1859.

**Common name.** Natal bubble shell.

**Size.** Maximum shell length 19 mm^(1)^.

**Remarks.** Inhabits shallow water including tidal rock pools^(34)^.

**Distribution.** On the South African coast, replaces *Haminoea alfredensis* from the Transkei into northern KwaZulu-Natal^(1)^. Occurs from Port Alfred to Mozambique and is likely synonymous with other Indo-Pacific species^(34)^.

**St Lucia records.** Probably misidentified in 1972–73^(25)^ as *Cylichna africana*, as misspelled *Haminea gracilis* in 1964–65^(21)^ and as misspelled *Haminea petersi* in 1981–82^(30)^. Reported in 1948–51^(20)^, as well as in 2006^(12)^ and 2007^(14)^. Only dead shells retrieved during the recent survey.

### Family: Aplysiidae

#### *Bursatella leachii* Blainville, 1817

**St Lucia synonyms.** *Barnardaclesia cirrhifera* (Quoy & Gaimard, 1832); *Notarchus cirrhifera* (Quoy & Gaimard, 1832).

**Common name.** Shaggy Sea Hare, Ragged Sea Hare.

**Size.** Maximum length 130 mm^(34)^.

**Remarks.** Common estuarine species which also occurs in tide pools^(19)^.

**Distribution.** Cicumtropical species reported from the east coast of Africa^(19)^, the Mediterranean Sea^(27)^, the North Atlantic region^(28)^ as well as the Caribbean^(35)^.

**St Lucia records.** Reported as *Barnardaclesia cirrhifera* in 1948–51^(20)^ and as *Notarchus cirrhifera* in 1964–65^(21)^. Not recorded during the recent survey.

#### *Stylocheilus striatus* (Quoy & Gaimard, 1832)

**Common name.** Lined Sea Hare.

**Size.** 28 mm^(33)^.

**Remarks.** Occurs in sheltered pools and estuaries, often sympatric with *Bursatella leachii*.

**Distribution.** Circumtropical distribution including Cape Verde^(36)^ and the Caribbean Sea^(35)^. Southern African distribution extends from Mngazana in the Eastern Cape to southern Mozambique^(34)^.

**St Lucia records.** Recorded in 2007^(14)^ after the overtopping and mouth breaching event. Not reported in the recent survey.

### Family: Siphonariidae

#### *Siphonaria oculus* Krauss, 1848

**Common name.** Eyed false-limpet^(19)^.

**Size.** Maximum length 33 mm^(1)^.

**Remarks.** Locally common species which is found on sheltered rocks in lagoons and estuaries^(1)^.

**Distribution.** Southern African distribution extends along the coast from False Bay in the Western Cape Province to Mozambique^(1)^.

**St Lucia records.** Reported in 1948–51^(20)^ and 1964–65^(21)^ from the St Lucia Mouth. Not recorded in the recent survey.

### Family: Planorbidae

#### *Bulinus natalensis* (Krauss in Küster, 1841)

**Common name.** Natal bladder snail

**Size.** 9.6 × 8.5 mm (depressed form) 9.5 × 6.5 mm (high spired form)^(2)^.

**Remarks.** Wide range of habitats including small pools, slow flowing rivers and lakes^(2)^.

**Distribution.** East African distribution extending from Ethiopia to the northern coastal region of KwaZulu-Natal^(2)^ where it occurs predominantly on the eastern lowlands^(24)^.

**St Lucia records.** Not previously reported from St Lucia; recorded in the recent survey at Catalina Bay (South Lake) in 2012.

#### *Bulinus tropicus* (Krauss, 1848)

**Common name.** Tropical bladder snail.

**Size.** 12.3 × 7.8 mm (slender form), 10.6 × 8.3 mm (more globose form)^(2)^.

**Remarks.** Commonly occurs in small earth dams and residual pools of seasonally flowing streams^(2)^.

**Distribution.** Eastern and southern Africa, extending from Ethiopia to Namibia and the Western Cape of South Africa. Not commonly found on the eastern coastal region of South Africa^(2)^.

**St Lucia records.** Reported in 1948^(20)^, 1964–65^(21)^ and 2002–03^(37)^. Only dead shells retrieved from the lake shores at Lister’s Point (False Bay), Hell’s Gate (North Lake), Fani’s Island (South Lake) and Mpate Mouth (Narrows) during the recent survey.

#### *Bulinus forskalii* (Ehrenberg, 1831)

**Common name.** Forskål’s bladder snail.

**Size.** 17 × 5.4 mm^(2)^.

**Remarks.** Ability to aestivate allows this species to commonly inhabit seasonal pools^(24)^.

**Distribution.** Afrotropical distribution extending south from the Egyptian Mediterranean region to Namibia^(2)^. Also reported from Madagascar^(2)^.

**St Lucia records.** Reported in 2002–03^(37)^ from the South Lake Eastern Shores. Not reported during the recent survey.

### Family: Physidae

#### *Aplexa marmorata* (Guilding, 1828)

**Common name.** Slender bladder snail^(7)^.

**Size.** 15 × 8 mm^(3)^.

**Remarks.** Alien species introduced from South America^(3)^. Commonly colonizes lentic waterbodies and the backwaters of rivers^(24)^. Recent work assigns this species to the genus “*Aplexa*” rather than “*Physa*”^(38)^.

**Distribution.** Occurs in isolated populations in KwaZulu-Natal, Mpumalanga and Limpopo^(24)^.

**St Lucia records.** Reported in 2005^(39)^ from a pan on the Western Shores (South Lake). Recorded in 2009^(13)^, 2010^(14)^ and in the recent survey in 2012 from Catalina Bay (South Lake).

### Family: Lymnaeidae

#### *Lymnaea natalensis* (Krauss, 1848)

**Common name.** Natal pond snail.

**Size.** Maximum shell height 25 mm^(2)^.

**Remarks.** Occurs in permanent streams^(2)^. Major intermediate host of the giant liver fluke, *Fasciola gigantica*^(3)^. Debated whether this species should be assigned to the genus *Radix*.

**Distribution.** East African distribution including the highlands of Ethiopia^(2)^. Southern range includes the Orange, Okavango and Zambezi Rivers^(24)^.

**St Lucia records.** Not previously reported from St Lucia; collected in 1982 (NPB). Reported in the recent survey from Catalina Bay (South Lake) in 2012.

#### *Pseudosuccinea columella* (Say, 1817)

**Common name.** Reticulate pond snail.

**Size.** 17 × 9 mm^(2)^.

**Remarks.** Alien species introduced from North America. Occurs on damp mud at the water-air interface^(24)^. Intermediate host for *Fasciola hepatica* and *Fasciola gigantica*^(24)^.

**Distribution.** Widely introduced to many areas, including Puerto Rico, Europe and New Zealand. First reported from Africa in 1944 from the Western Cape Province of South Africa^(2)^.

**St Lucia records.** Reported in 2009^(13)^ from freshwater seepage area in Catalina Bay (South Lake). Not recorded in the recent survey.

### Family: Succineidae

#### *Oxyloma patentissima* (Menke in Pfeiffer, 1853)

**Common name.** Twisted amber snail^(40)^.

**Size.** Maximum shell length 10 mm^(3)^.

**Remarks.** Typically occurs on emergent vegetation alongside water^(3)^.

**Distribution.** Southern African distribution includes Mozambique, northern Botswana and Zimbabwe^(17)^. Found on the KwaZulu-Natal coastal belt between Park Rynie and Lake Sibaya^(40)^.

**St Lucia records.** Reported in 2002–03^(37)^ from South Lake Eastern Shores. Not recorded in the recent survey.

### Family: Ellobiidae

#### *Cassidula labrella* (Deshayes, 1830)

**Common name.** Keeled coffee-bean snail.

**Size.** 12 × 7.5 mm^(2)^.

**Remarks.** Typically found on the surface of firm mud in mangroves and salt marshes^(24)^.

**Distribution.** East African coastal distribution from the Massawa region of the Red Sea to Port Elizabeth in South Africa^(2)^.

**St Lucia records.** Reported in 1964–65^(21)^ as well as during the recent survey at the mangroves near the St Lucia Estuary Mouth.

#### *Melampus lividus* (Deshayes, 1830)

**Common name.** Common coffee-bean snail^(40)^.

**Size.** Maximum shell height 18 mm^(40)^.

**Remarks.** Reported as *Melampus ordinarius* Melvill & Ponsonby, 1901 from deep vertical cracks in high level outcrops at Mission Rocks^(40)^.

**Distribution.** Tropical to subtropical Indian Ocean distribution extending from East London north along the KwaZulu-Natal coast^(40)^.

**St Lucia records.** Reported in 1964–65^(21)^. Not recorded in the recent survey.

#### *Melampus parvulus* Pfeiffer, 1856

**Common name.** Dwarf coffee-bean snail^(40)^.

**Size.** Maximum shell height 13 mm^(40)^.

**Remarks.** Found on firm mud in lagoons and estuaries where individuals form dense colonies^(40)^.

**Distribution.** Tropical Indo-Pacific distribution including Indian Ocean islands. South African distribution extends from KwaZulu-Natal to Port Alfred^(40)^.

**St Lucia records.** Not previously reported from St Lucia; recorded from the mangroves at Shark Basin (St Lucia Mouth) in the recent survey.

#### *Melampus semiaratus* Connolly, 1912

**Common name.** Half-grooved coffee-bean snail^(40)^.

**Size.** Maximum shell height 12 mm^(40)^.

**Remarks.** Mangrove species which occurs in the burrows of crabs up to a depth of 150 mm as well as on the surface of the mud^(24)^.

**Distribution.** East African distribution ranging from the Giuba River in Tanzania to the Umkomaas River on the southern coast of KwaZulu-Natal in South Africa^(2)^.

**St Lucia records.** Reported in St Lucia in 1964–65^(21)^ as well as in the recent survey from the mangroves at Shark Basin (St Lucia Mouth) in 2012.
